# Intercalation Ability of Novel Monofunctional Platinum Anticancer Drugs: A Key Step in Their Biological Action

**DOI:** 10.1021/acs.jcim.1c00430

**Published:** 2021-06-22

**Authors:** Daniele Veclani, Marilena Tolazzi, José P. Cerón-Carrasco, Andrea Melchior

**Affiliations:** † Dipartimento Politecnico di Ingegneria e Architettura (DPIA), Laboratori di Chimica, Università di Udine, via delle Scienze 99, 33100 Udine, Italy; ‡ Reconocimiento y Encapsulación Molecular, Universidad Católica San Antonio de Murcia (UCAM). Campus de los Jerónimos, 30107 Murcia, Spain

## Abstract

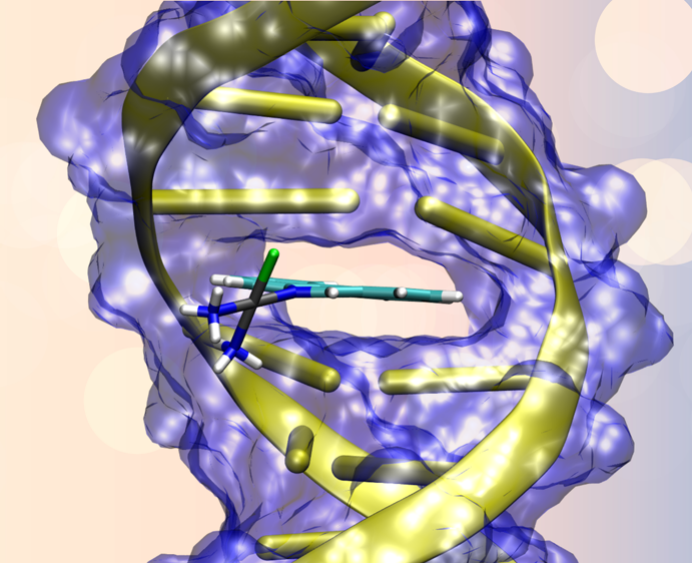

Phenanthriplatin (PtPPH) is a monovalent platinum(II)-based complex with a large cytotoxicity against cancer cells. Although the aqua-activated drug has been assumed to be the precursor for DNA damage, it is still under debate whether the way in which that metallodrug attacks to DNA is dominated by a direct binding to a guanine base or rather by an intercalated intermediate product. Aiming to capture the mechanism of action of PtPPH, the present contribution used theoretical tools to systematically assess the sequence of all possible mechanisms on drug activation and reactivity, for example, hydrolysis, intercalation, and covalent damage to DNA. *Ab initio* quantum mechanical (QM) methods, hybrid QM/QM′ schemes, and independent gradient model approaches are implemented in an unbiased protocol. The performed simulations show that the cascade of reactions is articulated in three well-defined stages: (i) an early and fast intercalation of the complex between the DNA bases, (ii) a subsequent hydrolysis reaction that leads to the aqua-activated form, and (iii) a final formation of the covalent bond between PtPPH and DNA at a guanine site. The permanent damage to DNA is consequently driven by that latter bond to DNA but with a simultaneous π–π intercalation of the phenanthridine into nucleobases. The impact of the DNA sequence and the lateral backbone was also discussed to provide a more complete picture of the forces that anchor the drug into the double helix.

## Introduction

After the discovery of the biological activity of cisplatin,^1^ great efforts have been made for the development of structurally similar bifunctional Pt(II) complexes to overcome the severe side effects^2,3^ and the tumor resistance over long-term treatments.^3^ Nowadays, besides cisplatin, only carboplatin and oxaliplatin are approved for clinical use worldwide.^4,5^ Other three platinum-based compounds (nedaplatin, lobaplatin, and heptaplatin) are approved in some countries only.^5^

The accepted mechanism of action of bifunctional platinum drugs involves the activation of the drug inside the cell by releasing a leaving ligand(s) and the reaction of the aqua-activated drug with DNA.^6^ This is followed by the formation of intra- and inter-strand cross-links which induce a distortion of the canonical DNA double helix which ultimately produces the cell death.^7^ Unfortunately, platinum drugs can also interact with other targets, such as metallo-proteins, responsible for the high side effects of these drugs.^8,9^

Monofunctional Pt(II) compounds might circumvent such limitations and improve the efficacy of the treatments.^5^ Among them, phenanthriplatin [*cis*-Pt(NH_3_)_2_(phenanthridine)Cl]^+^ (hereafter labeled as PtPPH) has been demonstrated to be active against 60 human cancer cell lines *in vitro* with a cytotoxicity even larger than that of the parent cisplatin.^10^ The proposed mechanism of action of these compounds differs from that reported for classical platinum-based drugs^11−14^ as PtPPH is able to bind to DNA to produce a single covalent bond. This type of adduct is not able to bend or unwind the double helix of the DNA; rather, it blocks the action of RNA and DNA polymerases with subsequent triggering of the processes which ultimately lead to apoptosis. Moreover, contrary to cisplatin, which specifically binds to the guanine base at the N7 position, PtPPH can react with both guanosine at the N7 site as well as with methyladenine at the N7 and N1 sites in a similar rate.^15^

To understand the molecular factors at play in such unique biological action, several theoretical studies focused on bifunctional^16−37^ and monofunctional^38−42^ Pt(II) complexes have been carried out. Nevertheless, as far as PtPPH is concerned, the mechanism of action is still under debate.

In a recent contribution aimed at replicating the experimental conditions and the mechanisms of reactions between PtPPH and DNA bases,^38^ we highlighted the possibility of formation of π–π interactions prior to the formation of the final covalent adduct. Recently, the DNA–PtPPH interaction was revised by Lippard and co-workers^43^ on the basis of the analysis of the time-dependent extensions of single λ-DNA molecules treated with the cis- and trans-isomers of PtPPH. The latter results suggested that the mechanism of binding involves a fast intercalation step which leads producing a stretching of the DNA, followed by a second slower reaction which is assigned to the covalent bond formation with the N7 atom of a purine base. It is remarkable that the mechanism is largely sensitive to the stereochemistry at the metal center as only the cis-isomer has the proper conformation to produce irreversible DNA elongation upon covalent bond formation. In the same work, a preliminary molecular docking of *cis*- and *trans*-PtPPH was performed. Although such a computational approach provides first clues about the intercalation phenomena, more refined models must be implemented if biological conclusions are looked for. In this framework, Dabbish^39,44^*et al.* have recently used density functional theory (DFT) calculations and classical molecular dynamics simulations to assess the hydrolysis, interaction with guanine (G), reactivity with *N*-acetyl methionine, and intercalation to DNA. However, this study has been exclusively focused on the reaction of the aqua-activated complex of PtPPH when analyzing the interaction with nucleobases so that it remains unknown the reactivity of the parent chloro complex.

The literature provides examples where intercalation is much faster than hydrolysis and subsequent covalent binding to DNA bases.^45−48^ If one brings such pieces of evidence to monovalent PtPPH, the chloro complex might be accumulated into the DNA double helix by “pure” intercalation at an early stage of the attack prior hydrolysis. This contribution aims to elucidate if this hypothesis also applies in the PtPPH case by assessing the impact of aqua-activation in the interaction of PtPPH with nucleobases. We designed a series of model systems of increasing complexity that are large enough to capture main interactions in DNA, for example, interbase hydrogen bonds (HBs) and π–π stacking interactions, while allowing to use quantum mechanical (QM) levels of theory.

## Models and Methods

### Chemical Systems

**Figure 1:**
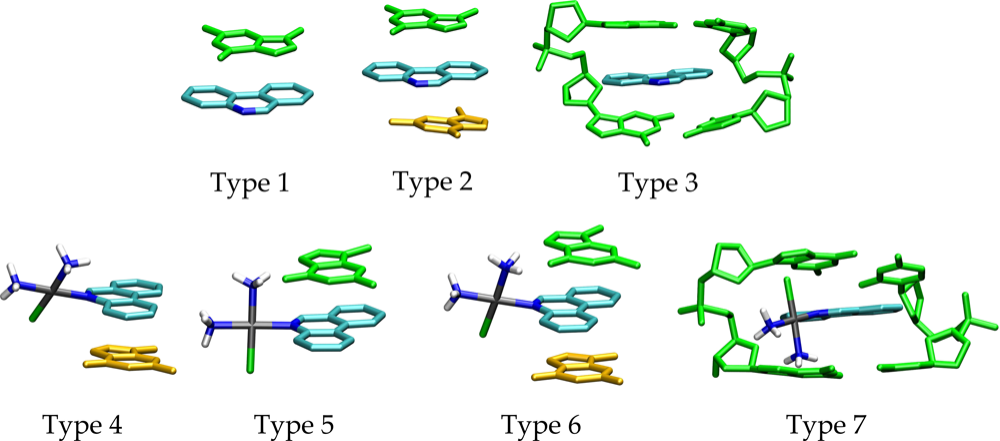
Types of clusters investigated: the top panel for the ligand and the bottom panel for the whole drug. In green yellow, top base and DNA; orange for the second base; in light blue, carbons; in blue, nitrogen; in gray, platinum atom.

The intercalation ability of the phenanthridine ligand (PPH) is first considered. As illustrated in the top panel of Figure 1, two minimal cluster models were built up to specifically account for the interaction of PPH with one or two free DNA bases, defined as types 1 and 2, respectively. Bases were used in the methylated forms, for example, 9-methyl-adenine (mA) and 9-methyl-guanine (mG). A larger DNA model (type 3) was next designed with a complete two-base-pair fragment, which also includes the lateral sugar-phosphate backbone. Guanine–cytosine (G–C) and adenine–thymine (A–T) positions were based on the standard double-stranded B-DNA form in the (5′ → 3′) CAACTAGCCGGT sequence.^49,50^ The sugar-phosphate backbone was protonated in order to obtain a neutral structure as recommended for mimicking these fragments with DFT methods.^51,52^

Similar DNA models were used for the chloro and aqua complexes with a single base interacting with different sides of the phenanthridine plane (types 4 and 5, bottom panel in Figure 1). In models labeled as type 6, two bases interact simultaneously with the phenanthridine ring. In these models, the interactions of Pt complexes were studied with two orientations of mA, (i) with the N1 atom (N1–mA) and (ii) with the N7 atom (N7–mA).

The type 7 counterpart includes two base pairs (G–C and A–T) which are connected by the deoxyribose phosphate linker. The latter models were employed to study the properties of PtPPH bound covalently to the N7 atom of G and A bases. Two possible orientations of the metal fragment have been studied, the first where the PPH ligand remains intercalated between the DNA bases (inside conformation, ins) and the second where this ligand is not intercalated and points away from the bases (outer conformation, out). The impact of the border base pairs in the intercalation mechanism has been tackled by defining several sequences, for example, A–T/A–T, A–T/G–C, and G–C/G–C intercalations sites, which have been used in earlier theoretical^53−56^ and experimental^48,57^ contributions.

### Computational Methods

DFT calculations were carried out using the B3LYP functional.^58−60^ Dispersion correction has been included employing the Grimme’s pair-wise additive method, DFT-D3.^61^ Previous works showed that the B3LYP-D3 method provides reliable results for structures, thermochemistry, and kinetic activation parameters for Pt(II) complexes.^38,39,62−65^ The Def2SVP basis set^66,67^ for all atoms was employed with relative effective core potential for the platinum atom. The solvent has been introduced as polarizable continuum (PCM).^68^ Frequency calculations were conducted for all located stationary points to confirm their nature of minima or transition states.

The more complete type 3 and 7 models are characterized by large electronic and geometrical degrees of freedom. A hybrid QM/QM′ scheme was used in these models as implemented in the ONIOM approach.^69,70^ The high layer (QM) included the intercalated platinum complexes, and two adjacent base pairs (four bases) were treated with the DFT level of theory described in the previous section. The low layer (QM′) was modeled with the semiempirical PM6 method.^71^ This QM/QM′ regime has been successfully used in the related biological system.^72−75^ To avoid unrealistic conformations, partial optimization was performed in three steps: first, the geometry was left to change without constraints; then, in a second stage, the positions of the all skeleton atoms (phosphate and deoxyribose) were frozen; in the third stage, the QM part was isolated, and with the optimized DFT level of theory described above, the coordinates of the four carbon atoms of the skeleton linked to the bases were frozen. All calculations were carried out with the Gaussian16 program.^76^

### Data Analysis

The interaction energy (IE) for two-body systems, for example, model types 1, 4, and 5, is defined through the regular eq 1
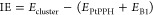
1where *E*_cluster_ is the total energy for the clusters, *E*_PtPPH_ is the energy of the isolated PtPPH drug, and *E*_B1_ represents the energy of the single DNA base. As shown in Figure 1, model type 1 accounts for the raw ligand PPH instead of the whole PtPPH so that in this case, the *E*_PtPPH_ term corresponds to the energy of the ligand. For the sake of clarity, hereafter, we decided to light notation by specifying in all cases *E*_PtPPH_. As far as model types 2 and 6 are concerned, the IE is consistently defined by eq 2
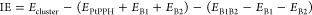
2where *E*_B1B2_ is the energy of the dimer formed by the two bases. Formally, the last term in eq 2 measures the mutual interaction of the two border bases. Because of the effect of the intercalated drug and the concomitant expansion into the base pair step distance, such energy results negligible in all cases (∼0.3 kcal mol^–1^). In the case of types 3 and 7, the computed IE depends on the energy of the two two base pairs, which is defined as *E*_2bp_ in eq 3
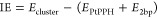
3

All IEs have been corrected (IE_Corr_) by adding the basis set superposition error using the counterpoise method by Boys and Bernardi.^77^ All energies used along with the discussion are given after such correction.

The quantitative study of the DNA–drug interaction is completed with a qualitative analysis performed by means of the recently developed independent gradient model (IGM) method, which is the natural evolution of traditional non-covalent interaction descriptors.^78,79^ IGM computes a new δ*g* parameter that measures the difference between a non-interacting model (the IGM), represented by a virtual upper limit of the electron density gradient (|∇ρ^IGM^|), and the real system, represented by the true electron density gradient (|∇ρ|). IGM analysis is implemented in our protocol to further identify the inter-fragment interactions that govern the stability of resulting drug–DNA clusters.

## Results and Discussion

As stated above, most of the recent computational efforts have been devoted to simulate the attack of the activated PtPPH toward DNA. Herein, all available mechanisms are investigated. We initially discuss the interaction of the used raw ligand in this metallodrug with a minimal DNA model, which is gradually completed with larger chemical motifs. Chemical systems are divided as follows: (i) PPH ligand versus DNA models, (ii) the whole PtPPH metallodrug with isolated DNA bases, (iii) the PtPPH intercalation mode into a DNA double helix, (iv) the release of the chloride ligand upon hydrolysis, and (v) intercalation of PtPPH covalently bound to G and A N7. The main text summarized our theoretical outcomes. The reader is referred to the Supporting Information for additional numeric results and structural data.

### Interaction of PPH Ligand DNA

**Figure 2:**
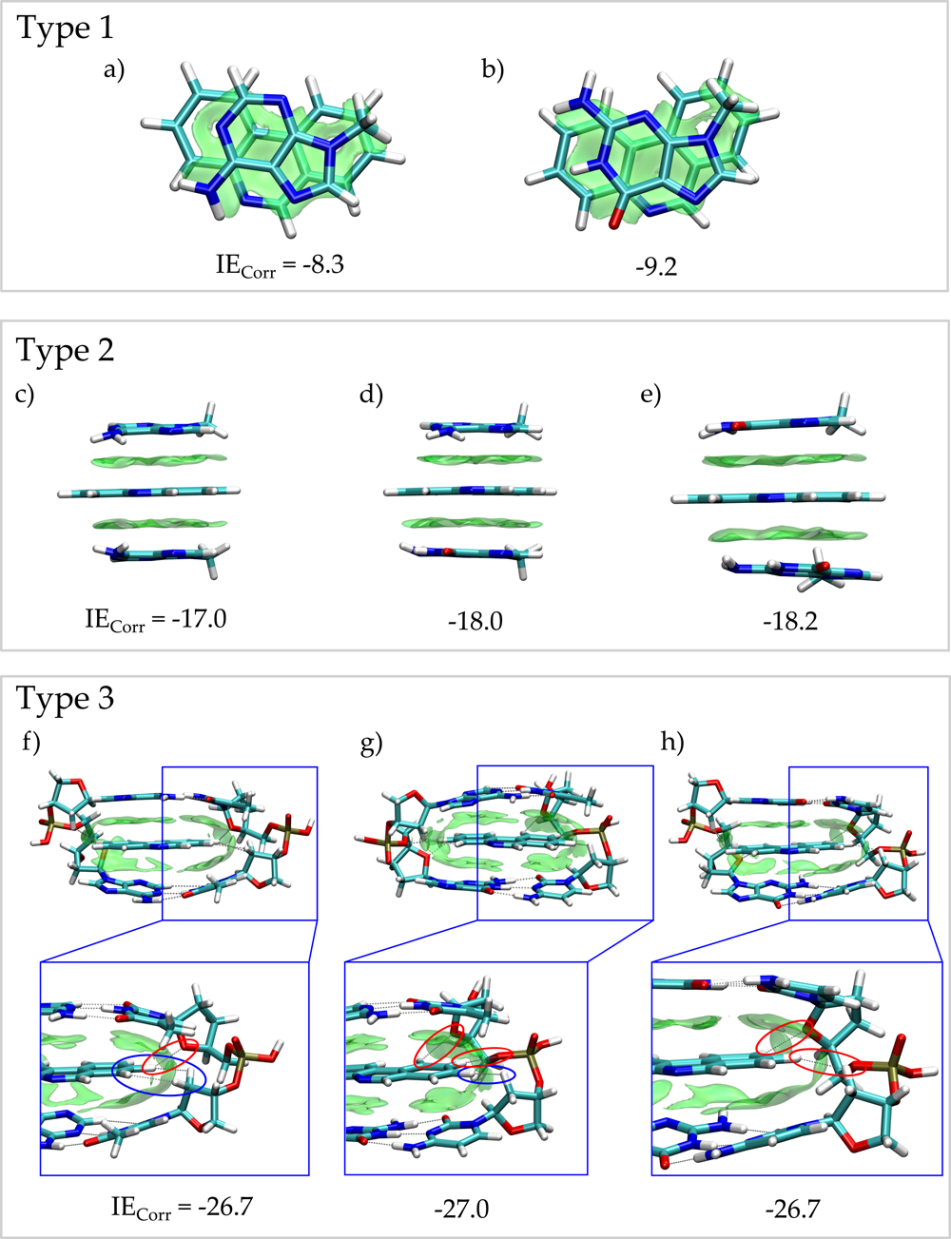
Minimum energy structures with the color-filled δ*g*^inter^ surfaces (isovalue 0.0055 a.u.) and relative IE_Corr_ for type 1 clusters (a) PPH–mA and (b) PPH–mG (top view); type 2 (c) mA–PPH–mA, (d) mAG–PPH–mA, and (e) mG–PPH–mG; type 3 (f) A–PPH–A, (g) G–PPH–A, and (h) G–PPH–G. CH–H interactions are highlighted in blue, and CH–O interactions are highlighted in red. IE_Corr_ in kcal mol^–1^.

The intercalation ability of an isolated PPH ligand is first delineated. To this end, the π–π interaction between the aromatic moieties of the ligand with one or two DNA bases is predicted by using model types 1 and 2, respectively (Figure 1, top panel). These results are summarized in Figure 2 and Table 1 (see also Table S1). As expected, in the absence of any other perturbative entity, all located structures correspond to a parallel orientation between bases and PPH due to π–π interactions. IGM analysis localizes such non-covalent interactions as two sharp spikes at low-δ*g*^inter^ values (see also Figure S1a,b).

**Table 1:** IE_Corr_ (kcal mol^–1^) for Cluster Types 1–3 Calculated in Water in Figure 1^a^

cluster type	sequence	IE_Corr_
1	PPH–mA	–8.3
	PPH–mG	–9.2
2	mA–PPH–mA	–17.0
	mG–PPH–mA	–18.0
	mG–PPH–mG	–18.2
3	A–PPH–A	–26.7
	G–PPH–A	–27.0
	G–PPH–G	–26.7

a More details are reported in Table S1.

According to the energies listed in Table 1, cluster types 1 and 2 show a slightly stronger interaction (∼1 kcal mol^–1^) when mG is present in the cluster. This conclusion is also supported by all possible orientations (Figure S2). It should be noted that the computed IE_Corr_ for PPH-base lies in the same range compared to energies reported for the base–base interactions^80−82^ so that this ligand is assembled in DNA at least as efficient as natural bases.

In type 3 clusters, PPH was intercalated into two base pairs connected by the sugar-phosphate linker (Figure 2). Of course, a larger π–π interaction is detected in cluster type 3 compared to the single-base models, which in turn yields to more negative IE_Corr_ values. Figure 2 (see also Figure S1c) demonstrates that the presence of the two further bases, thymine and cytosine, and the presence of the backbone interact with PPH through π–π, CH–H (Figure 2 highlighted in blue), and CH–O (Figure 2 highlighted in red) interactions. Consistent results were recently obtained by Gil and co-workers for a series of phenanthroline derivatives.^83^

The observed IE_Corr_ differences were minimal and suggest that PPH has a similar stacking preference for both mA- and mG-rich regions. In addition, theory foresees that while the energetic contribution of the π–π interaction was about −9 kcal mol^–1^ per base, the presence of the complete DNA base pairs and the lateral backbone (type 3) leads to an additional energetic contribution of −9 kcal mol^–1^. Consequently, although minimal models (i.e., isolated bases) arise as useful models to individually establish the contribution of stacking to the intercalation binding mode, all macroscopic conclusions should be extracted from the complete fragment.

### PtPPH Interaction with Isolated DNA Bases

**Figure 3:**
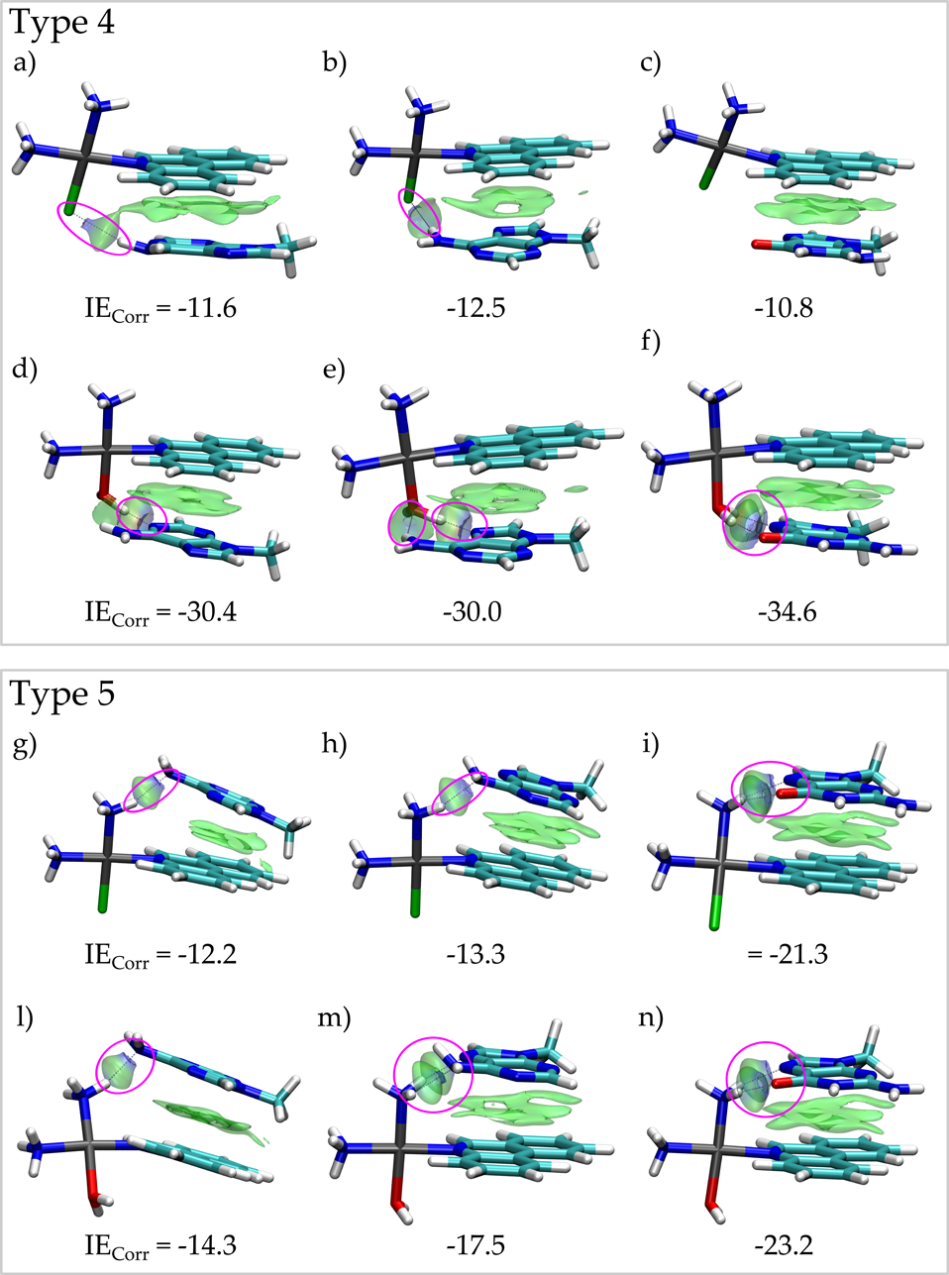
Minimum energy structures with the color-filled δ*g*^inter^ surfaces (isovalue 0.0055 a.u.) for type 4 clusters (a) NH_3_–PtPPH–Cl–N1–mA, (b) NH_3_–PtPPH–Cl–N7–mA, (c) NH_3_–PtPPH–Cl–N7–mG, (d) NH_3_–PtPPH–H_2_O–N1–mA, (e) NH_3_–PtPPH–H_2_O–N7–mA, and (f) NH_3_–PtPPH–H_2_O–N7–mG. Type 5 (g) mA–N1–NH_3_–PtPPH–Cl, (h) mA–N7–NH_3_–PtPPH–Cl, (i) mG–N7–NH_3_–PtPPH–Cl, (l) mA–N1–NH_3_–PtPPH–H_2_O, (m) mA–N7–NH_3_–PtPPH–H_2_O, and (n) mG–N7–NH_3_–PtPPH–H_2_O. HBs are circled in magenta. IE_Corr_ in kcal mol^–1^.

We adopted a similar computational strategy for addressing the intercalation ability of the whole chloro- and aqua-activated PtPPH complexes. Let us start with the intercalation ability with the single bases, for example, model types 4 and 5 (Figure 1). Listed energies in Table 2 (see also Table S2) show that the inactive PtPPH, that is, the form with a chloride ligand coordinated to the metallic center, leads to stronger interactions with type 5 clusters than with type 4 structures (Figure 3).

**Table 2:** IE_Corr_ (kcal mol^–1^) Obtained in Water for the Clusters Containing PtPPH and Aqua-PtPPH^a^

type	PtPPH	IE_Corr_	aqua-PtPPH	IE_Corr_
4	PtPPH–N1–mA	–11.6	PtPPH–N1–mA	–30.4
	PtPPH–N7–mA	–12.5	PtPPH–N7–mA	–30.0
	PtPPH–N7–mG	–10.8	PtPPH–N7–mG	–34.6
5	mA–N1–PtPPH	–12.2	mA–N1–PtPPH	–14.3
	mA–N7–PtPPH	–13.3	mA–N7–PtPPH	–17.5
	mG–N7–PtPPH	–21.3	mG–N7–PtPPH	–23.2
6	mA–N1–PtPPH–N1–mA	–29.6	mA–N1–PtPPH–N1–mA	–47.1
	mA–N7–PtPPH–N1–mA	–28.4	mA–N7–PtPPH–N1–mA	–46.2
	mG–N7–PtPPH–N1–mA	–33.7	mG–N7–PtPPH–N1–mA	–48.9
	mA–N1–PtPPH–N7–mA	–30.5	mA–N1–PtPPH–N7–mA	–46.9
	mA–N7–PtPPH–N7–mA	–28.9	mA–N7–PtPPH–N7–mA	–46.2
	mG–N7–PtPPH–N7–mA	–34.1	mG–N7–PtPPH–N7–mA	–48.1
	mA–N1–PtPPH–N7–mG	–29.5	mA–N1–PtPPH–N7–mG	–51.7
	mA–N7–PtPPH–N7–mG	–27.1	mA–N7–PtPPH–N7–mG	–47.3
	mG–N7–PtPPH–N7–mG	–31.0	mG–N7–PtPPH–N7–mG	–53.0

a Models are defined in Figures 2, 3, S6, and S7.

According to our model definition and axis criteria, this outcome indicates that the non-activated drug stacks in a similar way when mA is present thanks to the formation of NH_2_–Cl (in type 3) and NH_2_–NH_3_ (in type 4 and Figure S3) HB. A decrease in IE_Corr_ value was observed in the type 4 model when two molecules of mG are present thanks to the formation of HBs between NH_3_ and the O/N7 atom of mG (Figures 3i and S3b). An opposite behavior is observed for the aqua-PtPPH form as the most favorable structure is observed for the stacking at the bottom plane (type 4) thanks to the stronger HB between H_2_O and mA or mG (Figures 3 and S4). In both models, the most intense interactions are reached with the mG base.

Contrary to the raw PPH ligand, the whole PtPPH drugs are suggested to have preference for guanine regions. This conclusion is consistent with both models. The dissimilarity in the top/bottom preference is due to a combined effect of the HBs with the coordinated water molecule and the DNA base and the increased charge of the drug after activation (chloride is a negative ligand, while water acts as a neutral group), which is confirmed through the IGM analysis (Figures S3 and S4).

The results derived from model types 4 and 5 highlight the impact of the activation in the stacking phenomena. In model type 6, the platinum complexes are sandwiched between two bases so that we observe the accumulation of non-covalent interactions at both top and bottom planes (Figures S5 and S6). In such a more complex scenario, the energetic pattern previously obtained with model types 4 and 5 is still retained. Energies depicted in Table 2 (see also Table S2) confirm that the strongest interactions are found if mG–N7 is located at the bottom (chloride form) and top (aqua-activated drug) planes. Again, the activated form leads to a more stable intercalation, as confirmed by IE_Corr_ and the non-covalent contacts detected by IGM (Figures S7 and S8).

The systematic analysis of the PPH complexes with mG and mA shows that (i) mG has a slightly stronger interaction with PPH with respect to mA; (ii) HBs strongly stabilize the clusters formed with PtPPH with respect to those with the PPH ligand, where only the π–π interactions are present; (iii) the charge of the complex has a notable effect on the IE_corr_; (iv) the presence of mG in cluster types 4–6 results in more negative IE when it can interact with H_2_O or NH_3_.

### PtPPH Intercalation into the Double Helix

A step further toward a more realistic model is made by considering the intercalation of PtPPH and aqua-PtPPH into two base pairs connected by the sugar-phosphate linker (type 7, Figure S9). A comparison of energies listed in Tables 2 and 3 shows that the lateral backbone stabilizes the formed drug–DNA adduct by ca. 5–10 kcal mol^–1^. This series of non-covalent interactions is also resolved by IGM (see Figures S9 and S10). The intercalation of the parent PtPPH (chloride form) results in a π–π stacking concomitant with the formation of non-covalent bonds between the NH_3_ and chloride ligands with the N7–G and N7–A atoms, respectively, while the replacement of the Cl ligand with H_2_O leads to larger number of HBs (Figures S9 and S10) with a consequent decrease in the IE_Corr_ values depicted in Table 3 (see also Table S3).

**Table 3:** IE_Corr_ (kcal mol^–1^) and the Increase in the Distance between the N7 Atoms of Two Successive Bases (Δ*d*_N7–N7_, in Percentage) as Defined in Type 7 Clusters

sequence	IE_Corr_	Δ*d*_N7–N7_
PtPPH		
A–N7–PtPPH–N7–A	–32.7	40
A–N7–PtPPH–N7–G	–37.4	46
G–N7–PtPPH–N7–A	–42.8	48
G–N7–PtPPH–N7–G	–38.9	35
Aqua-PtPPH		
A–N7–PtPPH–N7–A	–59.3	50
A–N7–PtPPH–N7–G	–60.8	40
G–N7–PtPPH–N7–A	–59.0	48
G–N7–PtPPH–N7–G	–59.5	36

The intercalation into DNA produces a local extension between adjacent base pairs, a parameter that might be used to monitor the impact of the drug in the double-helix architecture.^84,85^ This induced expansion has been determined by determining the distance between the N7 atoms belonging two successive G or A bases in type 7 clusters (*d*_N7–N7_). The DNA structure without the complex is used as a reference value. The optimized models exhibit a 35–50% increase in the *d*_N7–N7_ distance upon PtPPH intercalation, which agrees with the experimentally measured structure and confirms the accuracy of our computational protocol to reproduce DNA damage.^43^ This step-by-step modeling protocol allows us to determine that the presence of the lateral backbone does not reduce the intercalation ability of the drug but enables a more stable interaction with DNA by means of additional non-covalent contacts.

### Activation upon Hydrolysis

Pt-based drugs are injected as chloride derivatives so that their activation is a critical step in their biological activity.^6^ The natural gradient of chloride, which is present at a very high concentration in blood (Cl^–^ = 116 mM) but drastically decays inside the cell (Cl^–^ = 4 mM), eventually promotes the hydrolysis processes in the intracellular medium.^86^

**Figure 4:**
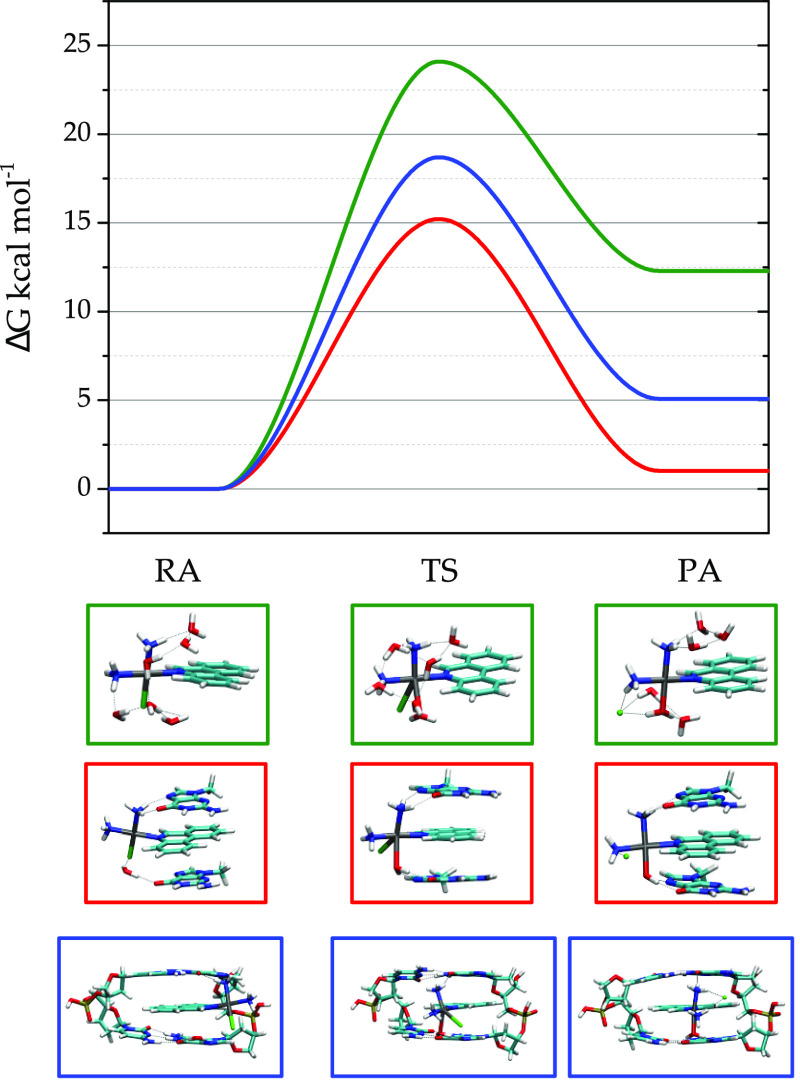
Computed free-energy profiles of the hydrolysis reactions: PtPPH with a hybrid explicit/implicit solution model (green line), intercalated to two free mG bases (red line, cluster type 6), and intercalated to a DNA dimer (GG) (blue line). The activation Δ*G*^‡^ [Δ*G*^‡^ = (*G*_TS_ – *G*_RA_)] and reaction Δ*G*_r_ free energies are reported in Table S4.

Aiming to determine whether activation occurs before or after intercalation, the type 6 model and the more complete type 7 model are employed to compute the energetic profile along with the release of the chloride ligand. The impact of stacking is predicted by including two additional models for PtPPH as a free entity, in which the drug is treated as a fully free/solvated PtPPH in water or located in the external region of DNA without intercalation (labeled as “out” conformation as a counterpart of the intercalated “ins” adducts). The structures of the reagents (RA), transition states (TS), and products (PA) are shown in Figure 4. For all TSs, the single-imaginary frequency confirms that obtained structures correspond to the rupture of the Pt–Cl bond and the simultaneous formation of the Pt–OH_2_ link.

The calculated free-energy profiles (Δ*G*) are also plotted in Figure 4. The activation energy for the free PtPPH entity (Δ*G*^‡^ = 24.1 kcal mol^–1^, Table S4) agrees with previous computational predictions by using implicit (PCM) and hybrid implicit/explicit water models.^38^ A close inspection of this figure shows that the intercalated PtPPH drugs undergo hydrolysis with a significant lower activation barrier (Δ*G*^‡^ = 18.7 kcal mol^–1^). Stacking of the PPH ligand and the additional contacts with the lateral backbone stabilize the TS associated to the chloride release, which in turn favors hydrolysis. Additionally, intercalation leads to a more thermodynamically stable product with respect to free PtPPH in water solution (Figure 4 and Table S4).

### Covalent Binding to DNA

**Figure 5:**
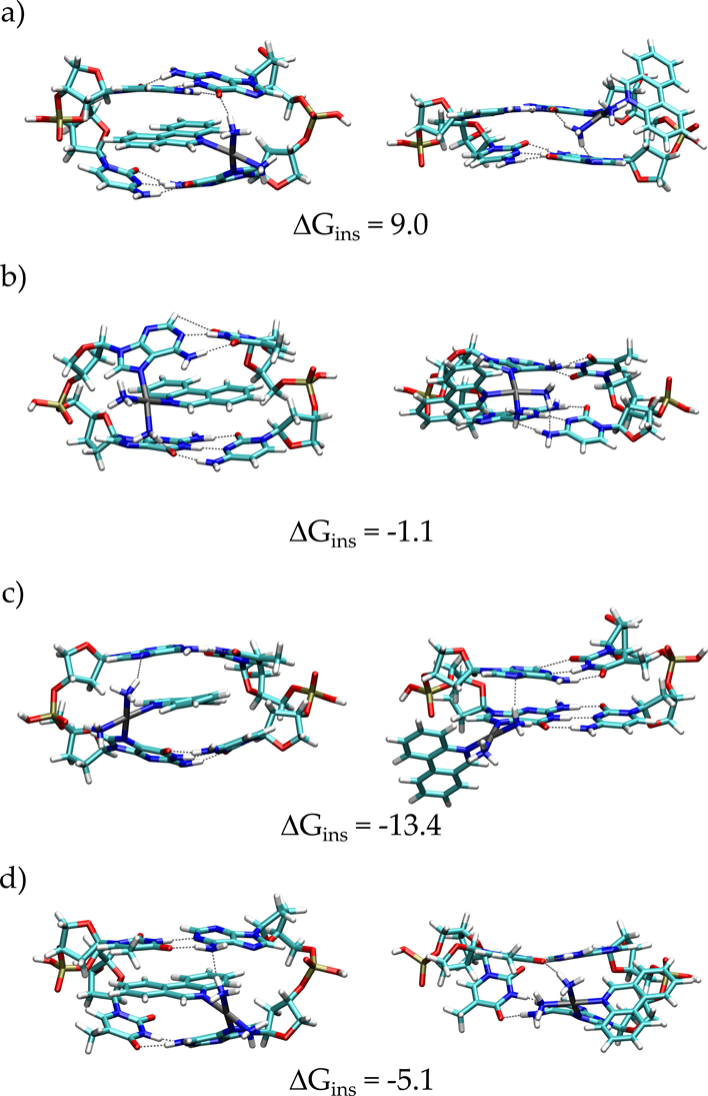
Minimum energy structures of the final products of the reaction of PtPPH with GA and GG dimers obtained by QM/QM′ calculations: (a) G–N7···NH_3_–PtPPH–N7–G, (b) A–N7···NH_3_–PtPPH–N7–G, (c) G–N7···NH_3_–PtPPH–N7–A, and (d) A–N7···NH_3_–PtPPH–N7–A with the complex in the intercalated and non-intercalated configurations. Δ*G*_ins_ (kcal mol^–1^) are also reported.

One remaining crucial issue needs to be addressed: the formation of the covalent bond with DNA at the reactive site in G and A bases, the so-called N7 positions. We used the activated drugs for assessing the structures of such a final step. The optimized structures are shown in Figure 5, which illustrates the combined intercalated/bound to DNA if the PPH ligand is stacked between base pairs, defined as “ins” conformation (see the hydrolysis section) in the top panel; the alternative activation without intercalation, where the drug remains “out” of DNA during activation, is also given in the bottom panel.

When PtPPH is bound to the GA dimers (Figure 5), the intercalated form (ins) results to be more stable than the non-intercalated one (out) as shown by the calculated Δ*G*_ins_ (=*G*_in_ – *G*_out_) equal to −1.1, −5.1, and −13.4 kcal mol^–1^ for the (b–c) adducts, respectively. On the contrary, in the case of coordination to the guanine N7 in the GG dimer, a positive Δ*G*_ins_ is found (9.0 kcal mol^–1^). Such dissimilarity correlates with the number of detected intra-molecular HBs: two HBs (NH_3_···O–G and NH_3_···N7–G) are observed in the out structure, although only one HB (NH_3_···O–G) is present in the ins counterpart (Figure 5a).

The analysis of the Δ*d*_N7–N7_ for the adducts in Figure 5 shows that when PPH is either intercalated or external to the bases, the lengthening (*d*_N7–N7_) is clearly lower (23–30% for the ins and 22–14% for the out) than that calculated for the type 7 clusters. These energetic and structural results agree with the coordination model of PtPPH where the DNA presents an irreversible lengthening in the final product.

## Conclusions and Outlook

PtPPH is a promising metallodrug based on a monovalent Pt(II) center. The attack to DNA is the ultimate step in the observed biological action. However, the cascade of reactions involved the interaction with the double-helix structure remains unclear. In this work, computational methods are used to assess the intercalation ability of the drug in the parent form as well as its aqua-activated drug. Several DNA models of increasing complexity are designed to delineate the impact of base pairs and the lateral backbone during reaction.

Our simulations with single-base models demonstrate that the used phenanthridine ligand (PPH) for decorating Pt(II) is fully compatible for stacking between DNA bases, with a π–π IE_corr_ similar to the natural bases. The coordination to the metallic center does not affect to such stacking ability. More elaborated DNA fragments, with a full sequence of two base pairs, show that PtPPH is able to intercalate into DNA as the stacking with PPH is the ligand, while additional contacts between the drug and the lateral DNA backbone further stabilize the adduct. The study reveals a preference for interacting with guanine-rich regions, which might be exploited for targeting specific genetic sequences. The study of the hydrolysis mechanism reveals that intercalated PtPPH shifts the equilibrium toward the aqua-activated drug form. The transition state associated to the release of the chloride ligand and the entrance of a water molecule into the sphere is also favored if a free PtPPH drug is considered as a reference. Stacking and interactions with the backbone consequently accelerate the hydrolysis of the drug.

The performed calculations help to complete the understanding of the mechanism of action of PtPPH, which might be articulated in three steps: (i) an initial intercalation of the complex between DNA bases, (ii) a quick hydrolysis reaction, and (iii) a final covalent binding to DNA. Although larger genetic sequences can be used in the future to refined studies of PtPPH,^87−91^ the performed calculations provide better insight into the chemical reactions that govern its biological action.

## Supporting Information Available

Optimized structures of the clusters not reported in the main article; IGM analysis results; IEs; and structures of the reagents, transition states, and products of the hydrolysis reactions along with the thermodynamic and kinetic parameters (PDF)

## Notes

The Cartesian coordinates of the minimum energy structures and transition states will be provided by the corresponding authors upon request. Software used for QM and QM/QM′ calculations: Gaussian16 Rev A.03 (https://gaussian.com/). Electron density analysis was carried out with IGMPlot version 2.4.2 (http://igmplot.univ-reims.fr/). Graphical visualization with VMD 1.9.3 (https://www.ks.uiuc.edu). All software is in the linux version.

## References

[ref1] Rosenberg B.; Van Camp L.; Krigas T. Inhibition of Cell Division in Escherichia coli by Electrolysis Products from a Platinum Electrode. Nature 1965, 205, 698–699. 10.1038/205698a0. 14287410

[ref2] Dasari S.; Bernard Tchounwou P. Cisplatin in Cancer Therapy: Molecular Mechanisms of Action. Eur. J. Pharmacol. 2014, 740, 364–378. 10.1016/j.ejphar.2014.07.025. 25058905 PMC4146684

[ref3] Kelland L. The Resurgence of Platinum-Based Cancer Chemotherapy. Nat. Rev. Cancer 2007, 7, 573–584. 10.1038/nrc2167. 17625587

[ref4] Wheate N. J.; Walker S.; Craig G. E.; Oun R. The Status of Platinum Anticancer Drugs in the Clinic and in Clinical Trials. Dalton Trans. 2010, 39, 8113–8127. 10.1039/c0dt00292e. 20593091

[ref5] Johnstone T. C.; Suntharalingam K.; Lippard S. J. The Next Generation of Platinum Drugs: Targeted Pt(II) Agents, Nanoparticle Delivery, and Pt(IV) Prodrugs. Chem. Rev. 2016, 116, 3436–3486. 10.1021/acs.chemrev.5b00597. 26865551 PMC4792284

[ref6] Jung Y.; Lippard S. J. Direct Cellular Responses to Platinum-Induced DNA Damage. Chem. Rev. 2007, 107, 1387–1407. 10.1021/cr068207j. 17455916

[ref7] Cerón-Carrasco J. P.; Jacquemin D.; Cauët E. Cisplatin Cytotoxicity: A Theoretical Study of Induced Mutations. Phys. Chem. Chem. Phys. 2012, 14, 12457–12464. 10.1039/c2cp40515f. 22495492

[ref8] Casini A.; Reedijk J. Interactions of Anticancer Pt Compounds with Proteins: An Overlooked Topic in Medicinal Inorganic Chemistry?. Chem. Sci. 2012, 3, 3135–3144. 10.1039/c2sc20627g.

[ref9] Janoš P.; Spinello A.; Magistrato A. All-Atom Simulations to Studying Metallodrugs/Target Interactions. Curr. Opin. Chem. Biol. 2021, 61, 1–8. 10.1016/j.cbpa.2020.07.005. 32781390

[ref10] Park G. Y.; Wilson J. J.; Song Y.; Lippard S. J. Phenanthriplatin, a Monofunctional DNA-Binding Platinum Anticancer Drug Candidate with Unusual Potency and Cellular Activity Profile. Proc. Natl. Acad. Sci. U.S.A. 2012, 109, 11987–11992. 10.1073/pnas.1207670109. 22773807 PMC3409760

[ref11] Wang D.; Zhu G.; Huang X.; Lippard S. J. X-Ray Structure and Mechanism of RNA Polymerase II Stalled at an Antineoplastic Monofunctional Platinum-DNA Adduct. Proc. Natl. Acad. Sci. U.S.A. 2010, 107, 9584–9589. 10.1073/pnas.1002565107. 20448203 PMC2906855

[ref12] Kellinger M. W.; Park G. Y.; Chong J.; Lippard S. J.; Wang D. Effect of a Monofunctional Phenanthriplatin-DNA Adduct on RNA Polymerase II Transcriptional Fidelity and Translesion Synthesis. J. Am. Chem. Soc. 2013, 135, 13054–13061. 10.1021/ja405475y. 23927577 PMC3791135

[ref13] Gregory M. T.; Park G. Y.; Johnstone T. C.; Lee Y.-S.; Yang W.; Lippard S. J. Structural and mechanistic studies of polymerase bypass of phenanthriplatin DNA damage. Proc. Natl. Acad. Sci. U.S.A. 2014, 111, 9133–9138. 10.1073/pnas.1405739111. 24927576 PMC4078841

[ref14] Cerón-Carrasco J. P.; Jacquemin D. Tuning the Optical Properties of Phenanthriplatin: Towards New Photoactivatable Analogues. ChemPhotoChem 2017, 1, 504–512. 10.1002/cptc.201700090.

[ref15] Riddell I. A.; Johnstone T. C.; Park G. Y.; Lippard S. J. Nucleotide Binding Preference of the Monofunctional Platinum Anticancer-Agent Phenanthriplatin. Chem.—Eur. J. 2016, 22, 7574–7581. 10.1002/chem.201600236. 27111128 PMC4884344

[ref16] Alberto M. E.; Lucas M. F. A.; Pavelka M.; Russo N. The Second-Generation Anticancer Drug Nedaplatin: A Theoretical Investigation on the Hydrolysis Mechanism. J. Phys. Chem. B 2009, 113, 14473–14479. 10.1021/jp9056835. 19778071

[ref17] Melchior A.; Sánchez Marcos E.; Pappalardo R.; Martínez J. M. Comparative Study of the Hydrolysis of a Third- and a First-Generation Platinum Anticancer Complexes. Theor. Chem. Acc. 2011, 128, 627–638. 10.1007/s00214-010-0825-4.

[ref18] Reddy V. P. B.; Mitra I.; Mukherjee S.; Sengupta P. S.; Dodda S. R.; Moi S. C. A theoretical investigation on hydrolysis mechanism of biologically relevant Pt(II)/Pd(II) complexes with σ-donor and π-acceptor carrier ligand. Chem. Phys. Lett. 2016, 657, 148–155. 10.1016/j.cplett.2016.06.004.

[ref19] Dell’Anna M. M.; Censi V.; Carrozzini B.; Caliandro R.; Denora N.; Franco M.; Veclani D.; Melchior A.; Tolazzi M.; Mastrorilli P. Triphenylphosphane Pt(II) Complexes Containing Biologically Active Natural Polyphenols: Synthesis, Crystal Structure, Molecular Modeling and Cytotoxic Studies. J. Inorg. Biochem. 2016, 163, 346–361. 10.1016/j.jinorgbio.2016.08.006. 27557979

[ref20] Mitra I.; Reddy V. P. B.; Mukherjee S.; Linert W.; Moi S. C. Hydrolysis Theory Based on Density Functional Studies for Cytotoxic Pt(II) and Pd(II) Complexes With Benzimidazole Derivative. Chem. Phys. Lett. 2017, 678, 250–258. 10.1016/j.cplett.2017.04.065.

[ref21] Mukherjee S.; Reddy V. P. B.; Mitra I.; Linert W.; Moi S. C. Hydrolysis Mechanism of (N, N) Chelated Cytotoxic Pt/Pd(II)-Dichloro Complexes: A Theoretical Approach. Chem. Phys. Lett. 2017, 678, 241–249. 10.1016/j.cplett.2017.04.069.

[ref22] Ritacco I.; Al Assy M.; Abd El-Rahman M. K.; Fahmy S. A.; Russo N.; Shoeib T.; Sicilia E. Hydrolysis in Acidic Environment and Degradation of Satraplatin: A Joint Experimental and Theoretical Investigation. Inorg. Chem. 2017, 56, 6013–6026. 10.1021/acs.inorgchem.7b00945. 28452475

[ref23] Melchior A.; Martínez J. M.; Pappalardo R. R.; Sánchez Marcos E. Hydration of Cisplatin Studied by an Effective Ab Initio Pair Potential Including Solute-Solvent Polarization. J. Chem. Theory Comput. 2013, 9, 4562–4573. 10.1021/ct400433c. 26589171

[ref24] Melchior A.; Tolazzi M.; Martínez J. M.; Pappalardo R. R.; Sánchez Marcos E. Hydration of Two Cisplatin Aqua-Derivatives Studied by Quantum Mechanics and Molecular Dynamics Simulations. J. Chem. Theory Comput. 2015, 11, 1735–1744. 10.1021/ct500975a. 26574384

[ref25] Baik M.-H.; Friesner R. A.; Lippard S. J. Theoretical Study of Cisplatin Binding to Purine Bases: Why Does Cisplatin Prefer Guanine over Adenine?. J. Am. Chem. Soc. 2003, 125, 14082–14092. 10.1021/ja036960d. 14611245

[ref26] Raber J.; Zhu C.; Eriksson L. A. Theoretical Study of Cisplatin Binding to DNA: The Importance of Initial Complex Stabilization. J. Phys. Chem. B 2005, 109, 11006–11015. 10.1021/jp050057d. 16852341

[ref27] Costa L. A. S.; Hambley T. W.; Rocha W. R.; De Almeida W. B.; Dos Santos H. F. Kinetics and Structural Aspects of the Cisplatin Interactions With Guanine: A Quantum Mechanical Description. Int. J. Quantum Chem. 2006, 106, 2129–2144. 10.1002/qua.20979.

[ref28] Gao Y.; Zhou L. DNA Bindings of a Novel Anticancer Drug, Trans-PtCl_2_(Isopropylamine)(3-Picoline), and Kinetic Competition of Purine Bases With Protein Residues in the Bifunctional Substitutions: A Theoretical DFT Study. Theor. Chem. Acc. 2009, 123, 455–468. 10.1007/s00214-009-0557-5.

[ref29] Xu Z.; Zhou L. A DFT study of a novel oxime anticancer trans platinum complex: Monofunctional and bifunctional binding to purine bases. Int. J. Quantum Chem. 2011, 111, 1907–1920. 10.1002/qua.22488.

[ref30] Zhang D.; Zhou L. Theoretical Insight Into Pd(en)(H_2_O)_2_^2+^ Binding to Guanine Form [Pd(en)(guanine)_4_]^4+^: Kinetic Control and Thermodynamic Control. Comput. Theor. Chem. 2011, 967, 102–112. 10.1016/j.comptc.2011.03.051.

[ref31] Šebesta F.; Burda J. V. Study on Electronic Properties, Thermodynamic and Kinetic Parameters of the Selected Platinum(II) Derivatives Interacting With Guanine. J. Inorg. Biochem. 2017, 172, 100–109. 10.1016/j.jinorgbio.2017.04.006. 28448876

[ref32] Tai T. B.; Nhat P. V. A DFT Investigation on Interactions Between Asymmetric Derivatives of Cisplatin and Nucleobase Guanine. Chem. Phys. Lett. 2017, 680, 44–50. 10.1016/j.cplett.2017.05.028.

[ref33] Zhao J.; Wang D.; Xu G.; Gou S. Improve the Anticancer Potency of the Platinum(II) Complexes Through Functionalized Leaving Group. J. Inorg. Biochem. 2017, 175, 20–28. 10.1016/j.jinorgbio.2017.06.016. 28689065

[ref34] Zimmermann T.; Burda J. V. Cisplatin Interaction With Amino Acids Cysteine and Methionine From Gas Phase to Solutions With Constant pH. Interdiscip. Sci.: Comput. Life Sci. 2010, 2, 98–114. 10.1007/s12539-010-0094-x. 20640800

[ref35] Chen B.; Zhou L. Computational Study on Mechanisms of the Anticancer Drug: Cisplatin and Novel Polynuclear Platinum(II) Interaction With Sulfur-Donor Biomolecules and DNA Purine Bases. Comput. Theor. Chem. 2015, 1074, 36–49. 10.1016/j.comptc.2015.09.023.

[ref36] Lau J. K.-C.; Deubel D. V. Loss of Ammine From Platinum(II) Complexes: Implications for Cisplatin Inactivation, Storage, and Resistance. Chem.—Eur. J. 2005, 11, 2849–2855. 10.1002/chem.200401053. 15744707

[ref37] Deubel D. V. Factors Governing the Kinetic Competition of Nitrogen and Sulfur Ligands in Cisplatin Binding to Biological Targets†. J. Am. Chem. Soc. 2004, 126, 5999–6004. 10.1021/ja0499602. 15137764

[ref38] Veclani D.; Melchior A.; Tolazzi M.; Cerón-Carrasco J. P. Using Theory To Reinterpret the Kinetics of Monofunctional Platinum Anticancer Drugs: Stacking Matters. J. Am. Chem. Soc. 2018, 140, 14024–14027. 10.1021/jacs.8b07875. 30185041

[ref39] Dabbish E.; Russo N.; Sicilia E. Rationalization of the Superior Anticancer Activity of Phenanthriplatin: An In-Depth Computational Exploration. Chem.—Eur. J. 2020, 26, 259–268. 10.1002/chem.201903831. 31614021

[ref40] Li C.; Zhao X.; Liu W.; Yin F.; Hu J.; Zhang G.; Chen G. DNA Structural Distortions Induced by a Monofunctional Trinuclear Platinum Complex with Various Cross-Links Using Molecular Dynamics Simulation. J. Chem. Inf. Model. 2020, 60, 1700–1708. 10.1021/acs.jcim.0c00002. 32096984

[ref41] Dvořáčková O.; Chval Z. Tuning the Reactivity and Bonding Properties of Metal Square-Planar Complexes by the Substitution(s) on the Trans-Coordinated Pyridine Ring. ACS Omega 2020, 5, 11768–11783. 10.1021/acsomega.0c01161. 32478268 PMC7254792

[ref42] Hirakawa T.; Bowler D. R.; Miyazaki T.; Morikawa Y.; Truflandier L. A. Blue Moon Ensemble Simulation of Aquation Free Energy Profiles Applied to Mono and Bifunctional Platinum Anticancer Drugs. J. Comput. Chem. 2020, 41, 1973–1984. 10.1002/jcc.26367. 32590877

[ref43] Almaqwashi A. A.; Zhou W.; Naufer M. N.; Riddell I. A.; Yilmaz Ö. H.; Lippard S. J.; Williams M. C. DNA Intercalation Facilitates Efficient DNA-Targeted Covalent Binding of Phenanthriplatin. J. Am. Chem. Soc. 2019, 141, 1537–1545. 10.1021/jacs.8b10252. 30599508 PMC6491043

[ref44] Scoditti S.; Dabbish E.; Sicilia E. Is the cytotoxic activity of phenanthriplatin dependent on the specific size of the phenanthridine ligand π system?. J. Inorg. Biochem. 2021, 219, 111447 10.1016/j.jinorgbio.2021.111447. 33798829

[ref45] Nordell P.; Westerlund F.; Wilhelmsson L. M.; Nordén B.; Lincoln P. Kinetic Recognition of AT-Rich DNA by Ruthenium Complexes. Angew. Chem., Int. Ed. 2007, 46, 2203–2206. 10.1002/anie.200604294. 17310483

[ref46] D’Amico M. L.; Paiotta V.; Secco F.; Venturini M. A Kinetic Study of the Intercalation of Ethidium Bromide into Poly(A)-Poly(U). J. Phys. Chem. B 2002, 106, 12635–12641. 10.1021/jp025989l.

[ref47] Sischka A.; Toensing K.; Eckel R.; Wilking S. D.; Sewald N.; Ros R.; Anselmetti D. Molecular Mechanisms and Kinetics between DNA and DNA Binding Ligands. Biophys. J. 2005, 88, 404–411. 10.1529/biophysj.103.036293. 15516529 PMC1305017

[ref48] Furusawa H.; Nakayama H.; Funasaki M.; Okahata Y. Kinetic Characterization of Small DNA-binding Molecules Interacting With a DNA Strand on a Quartz Crystal Microbalance. Anal. Biochem. 2016, 492, 34–42. 10.1016/j.ab.2015.09.015. 26408811

[ref49] Shenoy S.; Jayaram B.; Latha N.; Narang P.; Jain T. P.; Bhushan K.; Shaikh S. A.; Bose S.; Sharma P.; Singhal P.; Gandhimathi A.; Agrawal P.; Pandey V.; Dutta S.; Sandhu G.; Gupta A.; Shekhar S.; Tripathi S. From Gene to Drug: A Proof of Concept for a Plausible Computational Pathway. Sixth International Conference on Intelligent Systems Design and Applications, 2006; Vol. 1, pp 1147–1152.

[ref50] Soni A.; Pandey K.; Ray P.; Jayaram B. Genomes to Hits In Silico - A Country Path Today, A Highway Tomorrow: A Case Study of Chikungunya. Curr. Pharm. Des. 2013, 19, 4687–4700. 10.2174/13816128113199990379. 23260020 PMC3831887

[ref51] Chung L. W.; Sameera W. M. C.; Ramozzi R.; Page A. J.; Hatanaka M.; Petrova G. P.; Harris T. V.; Li X.; Ke Z.; Liu F.; Li H.-B.; Ding L.; Morokuma K. The ONIOM Method and Its Applications. Chem. Rev. 2015, 115, 5678–5796. 10.1021/cr5004419. 25853797

[ref52] Langner K. M.; Janowski T.; Góra R. W.; Dziekoński P.; Sokalski W. A.; Pulay P. The Ethidium-UA/AU Intercalation Site: Effect of Model Fragmentation and Backbone Charge State. J. Chem. Theory Comput. 2011, 7, 2600–2609. 10.1021/ct200121f. 26606633

[ref53] Barone G.; Guerra C. F.; Gambino N.; Silvestri A.; Lauria A.; Almerico A. M.; Bickelhaupt F. M. Intercalation of Daunomycin into Stacked DNA Base Pairs. DFT Study of an Anticancer Drug. J. Biomol. Struct. Dyn. 2008, 26, 115–129. 10.1080/07391102.2008.10507229. 18533732

[ref54] Spinello A.; Terenzi A.; Barone G. Metal Complex-DNA Binding: Insights From Molecular Dynamics and DFT/MM Calculations. J. Inorg. Biochem. 2013, 124, 63–69. 10.1016/j.jinorgbio.2013.03.010. 23603013

[ref55] Elleuchi S.; De Luzuriaga I. O.; Sanchez-Gonzalez Á.; Lopez X.; Jarraya K.; Calhorda M. J.; Gil A. Computational Studies on the Binding Preferences of Molybdenum(II) Phenanthroline Complexes with Duplex DNA. The Important Role of the Ancillary Ligands. Inorg. Chem. 2020, 59, 12711–12721. 10.1021/acs.inorgchem.0c01793. 32806012

[ref56] Das S.; Roy S.; Bhattacharyya D. Dna Base Sequence Specificity Through Partial Intercalation: DFT-D Based Energy Analysis of Molecular Dynamics Snapshots. J. Mol. Graphics Modell. 2020, 101, 107722 10.1016/j.jmgm.2020.107722. 32882634

[ref57] Mårtensson A. K. F.; Abrahamsson M.; Tuite E. M.; Lincoln P. Diastereomeric Crowding Effects in the Competitive DNA Intercalation of Ru(phenanthroline)_2_dipyridophenazine^2+^ Enantiomers. Inorg. Chem. 2019, 58, 9452–9459. 10.1021/acs.inorgchem.9b01298. 31247836

[ref58] Becke A. D. Density-Functional Exchange-Energy Approximation With Correct Asymptotic Behavior. Phys. Rev. A: At., Mol., Opt. Phys. 1988, 38, 3098–3100. 10.1103/physreva.38.3098. 9900728

[ref59] Becke A. D. A new mixing of Hartree-Fock and local density-functional theories. J. Chem. Phys. 1993, 98, 1372–1377. 10.1063/1.464304.

[ref60] Lee C.; Yang W.; Parr R. G. Development of the Colle-Salvetti Correlation-Energy Formula Into a Functional of the Electron Density. Phys. Rev. B 1988, 37, 785–789. 10.1103/physrevb.37.785. 9944570

[ref61] Grimme S. Density Functional Theory With London Dispersion Corrections. Wiley Interdiscip. Rev.: Comput. Mol. Sci. 2011, 1, 211–228. 10.1002/wcms.30.

[ref62] Sukpattanacharoen C.; Kumar P.; Chi Y.; Kungwan N.; Escudero D. Formation of Excimers in Isoquinolinyl Pyrazolate Pt(II) Complexes: Role of Cooperativity Effects. Inorg. Chem. 2020, 59, 18253–18263. 10.1021/acs.inorgchem.0c02780. 33289543

[ref63] Nakagaki M.; Aono S.; Kato M.; Sakaki S. Delocalization of the Excited State and Emission Spectrum of the Platinum(II) Bipyridine Complex in Crystal: Periodic QM/MM Study. J. Phys. Chem. C 2020, 124, 10453–10461. 10.1021/acs.jpcc.9b11559.

[ref64] Endrizzi F.; Di Bernardo P.; Zanonato P. L.; Tisato F.; Porchia M.; Ahmed Isse A.; Melchior A.; Tolazzi M. Cu(i) and Ag(i) complex formation with the hydrophilic phosphine 1,3,5-triaza-7-phosphadamantane in different ionic media. How to estimate the effect of a complexing medium. Dalton Trans. 2017, 46, 1455–1466. 10.1039/c6dt04221j. 28074209

[ref65] Credendino R.; Minenkov Y.; Liguori D.; Piemontesi F.; Melchior A.; Morini G.; Tolazzi M.; Cavallo L. Accurate Experimental and Theoretical Enthalpies of Association of TiCl_4_ With Typical Lewis Bases Used in Heterogeneous Ziegler-Natta Catalysis. Phys. Chem. Chem. Phys. 2017, 19, 26996–27006. 10.1039/c7cp04047d. 28956566

[ref66] Weigend F.; Ahlrichs R. Balanced Basis Sets of Split Valence, Triple Zeta Valence and Quadruple Zeta Valence Quality for H to Rn: Design and Assessment of Accuracy. Phys. Chem. Chem. Phys. 2005, 7, 3297–3305. 10.1039/b508541a. 16240044

[ref67] Weigend F. Accurate Coulomb-fitting basis sets for H to Rn. Phys. Chem. Chem. Phys. 2006, 8, 1057–1065. 10.1039/b515623h. 16633586

[ref68] Tomasi J.; Mennucci B.; Cammi R. Quantum Mechanical Continuum Solvation Models. Chem. Rev. 2005, 105, 2999–3094. 10.1021/cr9904009. 16092826

[ref69] Dapprich S.; Komáromi I.; Byun K. S.; Morokuma K.; Frisch M. J. A New Oniom Implementation in Gaussian98. Part I. The Calculation of Energies, Gradients, Vibrational Frequencies and Electric Field Derivatives. J. Mol. Struct.: THEOCHEM 1999, 461–462, 1–21. 10.1016/s0166-1280(98)00475-8.

[ref70] Vreven T.; Byun K. S.; Komáromi I.; Dapprich S.; Montgomery J. A.; Morokuma K.; Frisch M. J. Combining Quantum Mechanics Methods with Molecular Mechanics Methods in ONIOM. J. Chem. Theory Comput. 2006, 2, 815–826. 10.1021/ct050289g. 26626688

[ref71] Stewart J. J. P. Optimization of Parameters for Semiempirical Methods V: Modification of NDDO Approximations and Application to 70 Elements. J. Mol. Model. 2007, 13, 1173–1213. 10.1007/s00894-007-0233-4. 17828561 PMC2039871

[ref72] Piazzetta P.; Marino T.; Russo N.; Salahub D. R. Explicit Water Molecules Play a Key Role in the Mechanism of Rhodium-Substituted Human Carbonic Anhydrase. ChemCatChem 2017, 9, 1047–1053. 10.1002/cctc.201601433.

[ref73] DeYonker N. J.; Webster C. E. A Theoretical Study of Phosphoryl Transfers of Tyrosyl-DNA Phosphodiesterase I (Tdp1) and the Possibility of a ″Dead-End″ Phosphohistidine Intermediate. Biochemistry 2015, 54, 4236–4247. 10.1021/acs.biochem.5b00396. 26121557

[ref74] Gesto D. S.; Cerqueira N. M. F. S. A.; Fernandes P. A.; Ramos M. J. Unraveling the Enigmatic Mechanism of L-Asparaginase II With QM/QM Calculations. J. Am. Chem. Soc. 2013, 135, 7146–7158. 10.1021/ja310165u. 23544711

[ref75] Galano A.; Alvarez-Idaboy J. R. On the Evolution of One-Electron-Oxidized Deoxyguanosine in Damaged DNA Under Physiological Conditions: A DFT and ONIOM Study on Proton Transfer and Equilibrium. Phys. Chem. Chem. Phys. 2012, 14, 12476–12484. 10.1039/c2cp40799j. 22644531

[ref76] Frisch M. J.; Trucks G. W.; Schlegel H. B.; Scuseria G. E.; Robb M. A.; Cheeseman J. R.; Scalmani G.; Barone V.; Petersson G. A.; Nakatsuji H.; Li X.; Caricato M.; Marenich A. V.; Bloino J.; Janesko B. G.; Gomperts R.; Mennucci B.; Hratchian H. P.; Ortiz J. V.; Izmaylov A. F.; Sonnenberg J. L.; Williams-Young D.; Ding F.; Lipparini F.; Egidi F.; Goings J.; Peng B.; Petrone A.; Henderson T.; Ranasinghe D.; Zakrzewski V. G.; Gao J.; Rega N.; Zheng G.; Liang W.; Hada M.; Ehara M.; Toyota K.; Fukuda R.; Hasegawa J.; Ishida M.; Nakajima T.; Honda Y.; Kitao O.; Nakai H.; Vreven T.; Throssell K.; Montgomery J. A. Jr.; Peralta J. E.; Ogliaro F.; Bearpark M. J.; Heyd J. J.; Brothers E. N.; Kudin K. N.; Staroverov V. N.; Keith T. A.; Kobayashi R.; Normand J.; Raghavachari K.; Rendell A. P.; Burant J. C.; Iyengar S. S.; Tomasi J.; Cossi M.; Millam J. M.; Klene M.; Adamo C.; Cammi R.; Ochterski J. W.; Martin R. L.; Morokuma K.; Farkas O.; Foresman J. B.; Fox D. J. Gaussian 16 Revision A.03; Gaussian Inc. Wallingford CT. 2016.

[ref77] Boys S. F.; Bernardi F. The Calculation of Small Molecular Interactions by the Differences of Separate Total Energies. Some Procedures With Reduced Errors. Mol. Phys. 1970, 19, 553–566. 10.1080/00268977000101561.

[ref78] Lefebvre C.; Khartabil H.; Boisson J.-C.; Contreras-García J.; Piquemal J.-P.; Hénon E. The Independent Gradient Model: A New Approach for Probing Strong and Weak Interactions in Molecules from Wave Function Calculations. ChemPhysChem 2018, 19, 724–735. 10.1002/cphc.201701325. 29250908

[ref79] Lefebvre C.; Rubez G.; Khartabil H.; Boisson J.-C.; Contreras-García J.; Hénon E. Accurately Extracting the Signature of Intermolecular Interactions Present In the NCI Plot of the Reduced Density Gradient Versus Electron Density. Phys. Chem. Chem. Phys. 2017, 19, 17928–17936. 10.1039/c7cp02110k. 28664951

[ref80] Czyżnikowska Ż. On the Importance of Electrostatics in Stabilization of Stacked Guanine-Adenine Complexes Appearing in B-DNA Crystals. J. Mol. Struct.: THEOCHEM 2009, 895, 161–167. 10.1016/j.theochem.2008.10.040.

[ref81] Šponer J.; Florián J.; Ng H. L.; Šponer J. E.; Špacková N. Local Conformational Variations Observed in B-DNA Crystals Do Not Improve Base Stacking: Computational Analysis of Base Stacking in a d(CATGGGCCCATG)(2) B<−>A Intermediate Crystal Structure. Nucleic Acids Res. 2000, 28, 4893–4902. 10.1093/nar/28.24.4893. 11121480 PMC115231

[ref82] Fonseca Guerra C.; van der Wijst T.; Poater J.; Swart M.; Bickelhaupt F. M. Adenine Versus Guanine Quartets in Aqueous Solution: Dispersion-Corrected DFT Study on The Differences in π-stacking and Hydrogen-Bonding Behavior. Theor. Chem. Acc. 2010, 125, 245–252. 10.1007/s00214-009-0634-9.

[ref83] Gil A.; Sanchez-Gonzalez A.; Branchadell V. Unraveling the Modulation of the Activity in Drugs Based on Methylated Phenanthroline When Intercalating between DNA Base Pairs. J. Chem. Inf. Model. 2019, 59, 3989–3995. 10.1021/acs.jcim.9b00500. 31419117

[ref84] Chantzis A.; Very T.; Daniel C.; Monari A.; Assfeld X. Theoretical Evidence of Photo-induced Charge Transfer from DNA to Intercalated Ruthenium (II) Organometallic Complexes. Chem. Phys. Lett. 2013, 578, 133–137. 10.1016/j.cplett.2013.05.068.

[ref85] Chantzis A.; Very T.; Despax S.; Issenhuth J.-T.; Boeglin A.; Hébraud P.; Pfeffer M.; Monari A.; Assfeld X. UV–Vis Absorption Spectrum of a Novel Ru(II) Complex Intercalated in DNA: [Ru(2,2’-bipy)(dppz)(2,2’-ArPy)]^+^. J. Mol. Model. 2014, 20, 2082–2092. 10.1007/s00894-014-2082-2. 24562852

[ref86] de Cózar A.; Larrañaga O.; Bickelhaupt F. M.; San Sebastián E.; Ortega-Carrasco E.; Maréchal J.-D.; Lledós A.; Cossío F. P. New Insights into the Reactivity of Cisplatin with Free and Restrained Nucleophiles: Microsolvation Effects and Base Selectivity in Cisplatin-DNA Interactions. ChemPhysChem 2016, 17, 3932–3947. 10.1002/cphc.201600982. 27642713

[ref87] Loos P.-F.; Dumont E.; Laurent A. D.; Assfeld X. Important Effects of Neighbouring Nucleotides on Electron Induced DNA Single-Strand Breaks. Chem. Phys. Lett. 2009, 475, 120–123. 10.1016/j.cplett.2009.05.041.

[ref88] Ambrosek D.; Loos P.-F.; Assfeld X.; Daniel C. Theoretical Study of Ru(II) Polypyridyl DNA Intercalators Structure and Electronic Absorption Spectroscopy of [Ru(phen)_2_(dppz)]^2+^ and [Ru(tap)_2_(dppz)]^2+^ Complexes Intercalated in Guanine–Cytosine Base Pairs. J. Inorg. Biochem. 2010, 104, 893–901. 10.1016/j.jinorgbio.2010.04.002. 20554006

[ref89] Cauët E.; Liévin J. Ab Initio Study of the Electron Transfer in an Ionized Stacked Complex of Guanines. J. Phys. Chem. A 2009, 113, 9881–9890. 10.1021/jp902426p. 19681582

[ref90] Cauët E.; Valiev M.; Weare J. H. Vertical Ionization Potentials of Nucleobases in a Fully Solvated DNA Environment. J. Phys. Chem. B 2010, 114, 5886–5894. 10.1021/jp9120723. 20394358

[ref91] Garrec J.; Patel C.; Rothlisberger U.; Dumont E. Insights into Intrastrand Cross-Link Lesions of DNA from QM/MM Molecular Dynamics Simulations. J. Am. Chem. Soc. 2012, 134, 2111–2119. 10.1021/ja2084042. 22200321

