# Supplementary material for: Intercalation Ability of Novel Monofunctional Platinum Anticancer Drugs: A Key Step in Their Biological Action
Source: J Chem Inf Model. 2021 Jun 22;61(9):4391–9. doi: 10.1021/acs.jcim.1c00430 (PMC8479807; doi:10.1021/acs.jcim.1c00430)
Supplement: Supplementary file 1 — ci1c00430_si_001.pdf [file ci1c00430_si_001.pdf]

Intercalation ability of novel monofunctional platinum anticancer drugs: a key step in their biological action

Daniele Veclani,[†] Marilena Tolazzi,[†] José Pedro Cerón-Carrasco[*‡] and Andrea Melchior[*†]

[†]Dipartimento Politecnico di Ingegneria e Architettura (DPIA), Laboratori di Chimica, Università di Udine, via delle Scienze 99 33100 Udine

[‡]Reconocimiento y Encapsulación Molecular, Universidad Católica San Antonio de Murcia (UCAM). Campus de los Jerónimos, 30107, Murcia, Spain.

Supporting Information

Table S1 IE, IE_{Corr} and BSSE in kcal mol^{-1} in gas phase and IE in water for PPH and type 1, 2 and 3 DNA model (see, Figure 1, top panel).

Cluster Type	Sequence	Gas Phase		BSSE	Water
		**IE**	**IE_{Corr}**		**IE**
1	PPH---mA	-14.1	-9.6	4.5	-12.8
	PPH---mG	-14.3	-9.6	4.7	-13.9
2	mA---PPH---mA	-28.4	-19.4	9.1	-26.0
	mG---PPH---mA	-28.4	-19.2	9.2	-27.2
	mG---PPH---mG	-32.1	-22.4	9.7	-28.3
3	A---PPH---A	-43.3	-29.3	14.0	-40.7
	G---PPH---A	-14.7	-43.3	14.7	-41.7
	G---PPH---G	-41.4	-27.2	14.3	-40.9

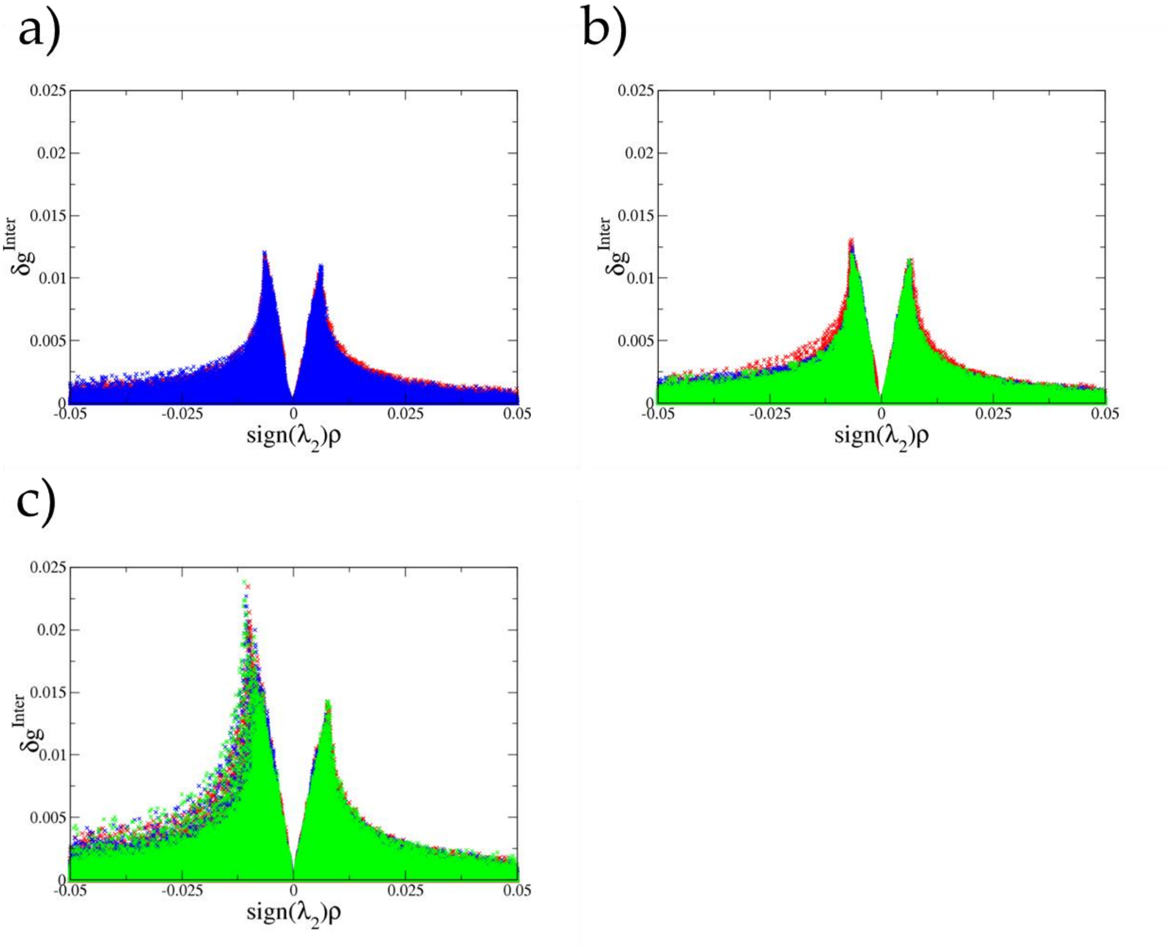

Figure S1 δg^{inter} plot for: a) PPH---mG (red, Figure 2a) and PPH---mA (blue, Figure 2b) Type 1 cluster. b) mA---PPH---mA (blue, Figure 2c), mG---PPH---mA (green, Figure 2d) and mG---PPH--m-G (red, Figure 2e) for Type 2 clusters. A---PPH---A (green, Figure 3a), A---PPH---G (blue, Figure 3b) and G---PPH---G (red, Figure 3c) Type 3 clusters.

Effect of Rotation of the base pairs

The effect of the relative orientation of the parallel base pairs on IE_{corr} was performed by a rigid scan of the dihedral angle defined by the atoms 1-2-3-4 in Figure S2 in the minimum energy structures of the Type 1 clusters.

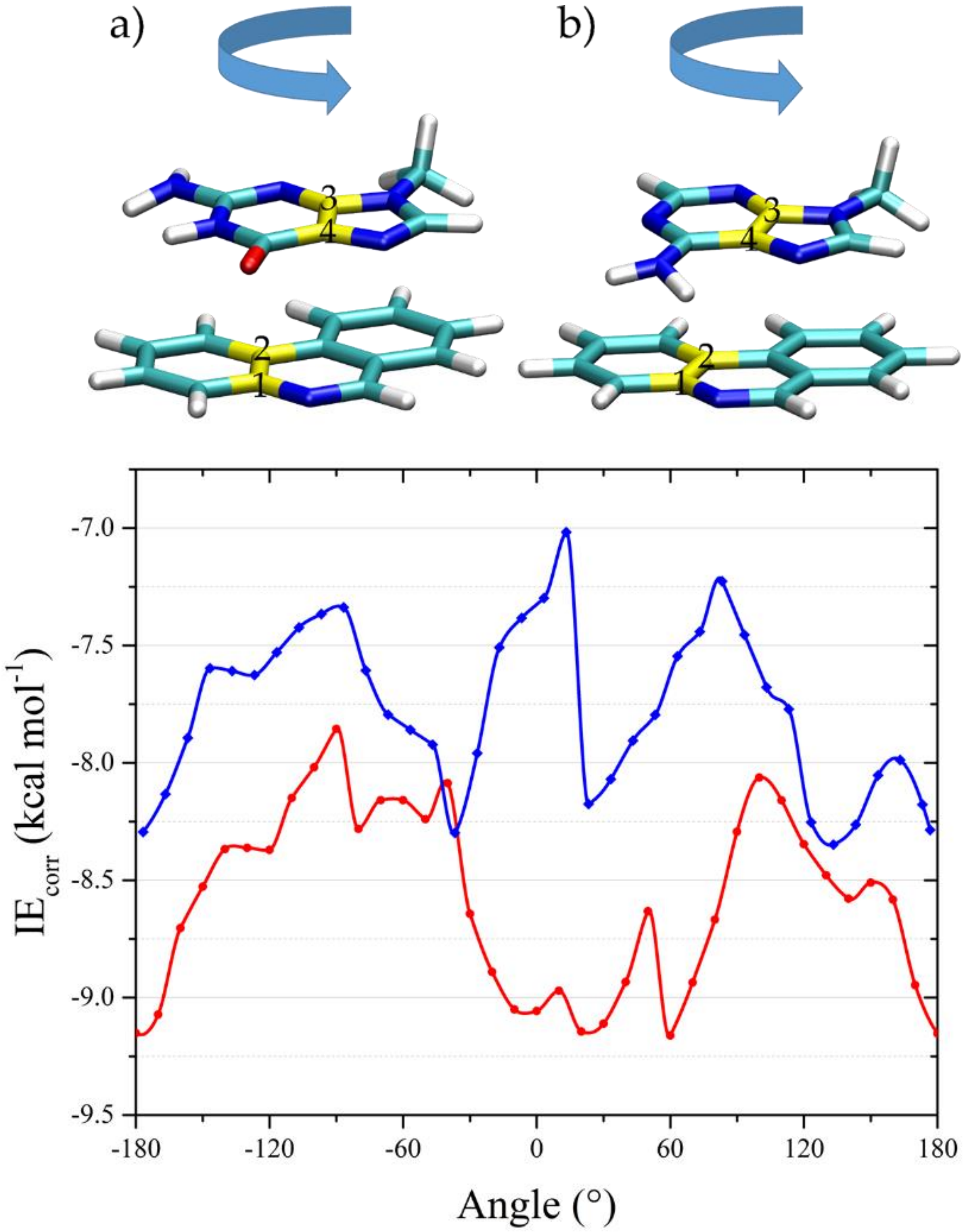

Figure S2 IE_{corr} *vs.* the value of the dihedral angle formed by the atoms 1-2-3-4, highlighted in yellow, for a) mG (red) and b) mA (blue).

Table S2 IE, IE_{Corr} and BSSE (kcal mol^{-1}) in gas phase and IE in water for PtPPH and *aqua*-PtPPH in Type 4, 5 and 6 models (Figure 4, 5, S5 and S6).

Cluster Type	Sequence	Gas Phase			Water
		**IE**	**IE_{Corr}**	**BSSE**	**IE**
	NH_3-PtPPH-Cl---N1-mA	-18.3	-13.3	5.0	-16.7
4	NH_3-PtPPH-Cl---N7-mA	-19.2	-13.8	5.4	-18.0
	NH_3-PtPPH-Cl---N7-mG	-18.4	-12.9	5.5	-16.3
	mA-N1---NH_3-PtPPH-Cl	-22.7	-18.1	4.6	-16.8
5	mA-N7---NH_3-PtPPH-Cl	-24.7	-19.5	5.2	-18.5
	mG-N7---NH_3-PtPPH-Cl	-48.0	-40.2	7.8	-29.1
	NH_3-PtPPH-H_2O---N1-mA	-56.4	-50.2	6.1	-36.5
4	NH_3-PtPPH-H_2O---N7-mA	-52.5	-45.8	6.7	-36.8
	NH_3-PtPPH-H_2O---N7-mG	-78.3	-69.9	8.4	-43.0
	mA-N1---NH_3-PtPPH-H_2O	-32.1	-27.2	4.9	-19.2
5	mA-N7---NH_3-PtPPH-H_2O	-42.0	-35.9	6.1	-23.6
	mG-N7---NH_3-PtPPH-H_2O	-64.1	-56.4	7.6	-30.9
	mA-N1---NH_3-PtPPH-Cl---N1-mA	-51.4	-40.2	11.2	-40.8
	mA-N7---NH_3-PtPPH-Cl---N1-mA	-47.7	-36.9	10.9	-39.3
	mG-N7---NH_3-PtPPH-Cl---N1-mA	-67.3	-54.7	12.6	-46.3
	mA-N7---NH_3-PtPPH-Cl---N7-mA	-52.0	-40.5	11.5	-42.0
6	mA-N1---NH_3-PtPPH-Cl---N7-mA	-47.7	-36.6	11.1	-40.0
	mG-N7---NH_3-PtPPH-Cl---N7-mA	-66.9	-54.1	12.8	-46.9
	mG-N7---NH_3-PtPPH-Cl---N7-mG	-49.5	-38.0	11.5	-41.0
	mA-N1---NH_3-PtPPH-Cl---N7-mG	-44.9	-34.0	10.9	-38.0
	mA-N7---NH_3-PtPPH-Cl---N7-mG	-63.0	-50.4	12.5	-43.5
	mA-N1---NH_3-PtPPH-H_2O---N1-mA	-90.6	-78.6	12.0	-59.1
	mA-N7---NH_3-PtPPH-H_2O---N1-mA	-86.9	-75.4	11.5	-57.7
	mG-N7---NH_3-PtPPH-H_2O---N1-mA	-108.2	-95.4	12.8	-61.7
	mA-N7---NH_3-PtPPH-H_2O---N7-mA	-88.0	-76.1	11.8	-58.7
6	mA-N1---NH_3-PtPPH-H_2O---N7-mA	-83.5	-71.6	11.9	-58.1
	mG-N7---NH_3-PtPPH-H_2O---N7-mA	-102.9	-89.7	13.3	-61.4
	mG-N7---NH_3-PtPPH-H_2O---N7-mG	-113.4	-99.4	14.0	-65.7
	mA-N1---NH_3-PtPPH-H_2O---N7-mG	-105.2	-92.5	12.7	-60.0
	mA-N7---NH_3-PtPPH-H_2O---N7-mG	-128.4	-113.5	14.9	-67.9

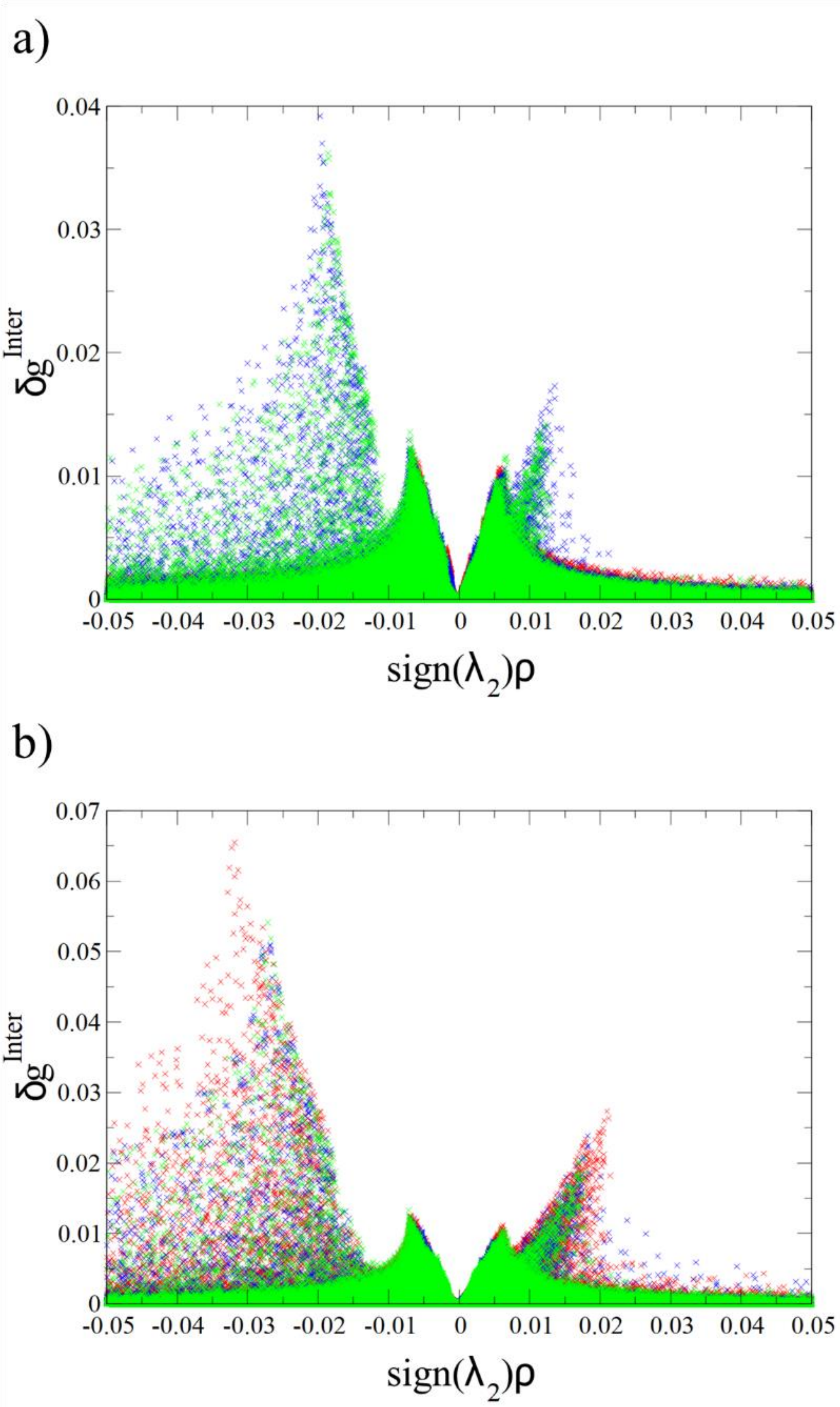

Figure S3 The δg^{inter} graph for a) Type 4 clusters (Figure 4a-c): NH_3-PtPPH-Cl---N7-mG (red), NH_3-PtPPH-Cl---N7-mA (blue) and NH_3-PtPPH-Cl---N1-mA (green). b) Type 5 clusters (Figure 4d-f): mG-N7---NH_3-PtPPH-Cl (red), mA-N7---NH_3-PtPPH-Cl (blue) and mA-N1---NH_3-PtPPH-Cl (green).

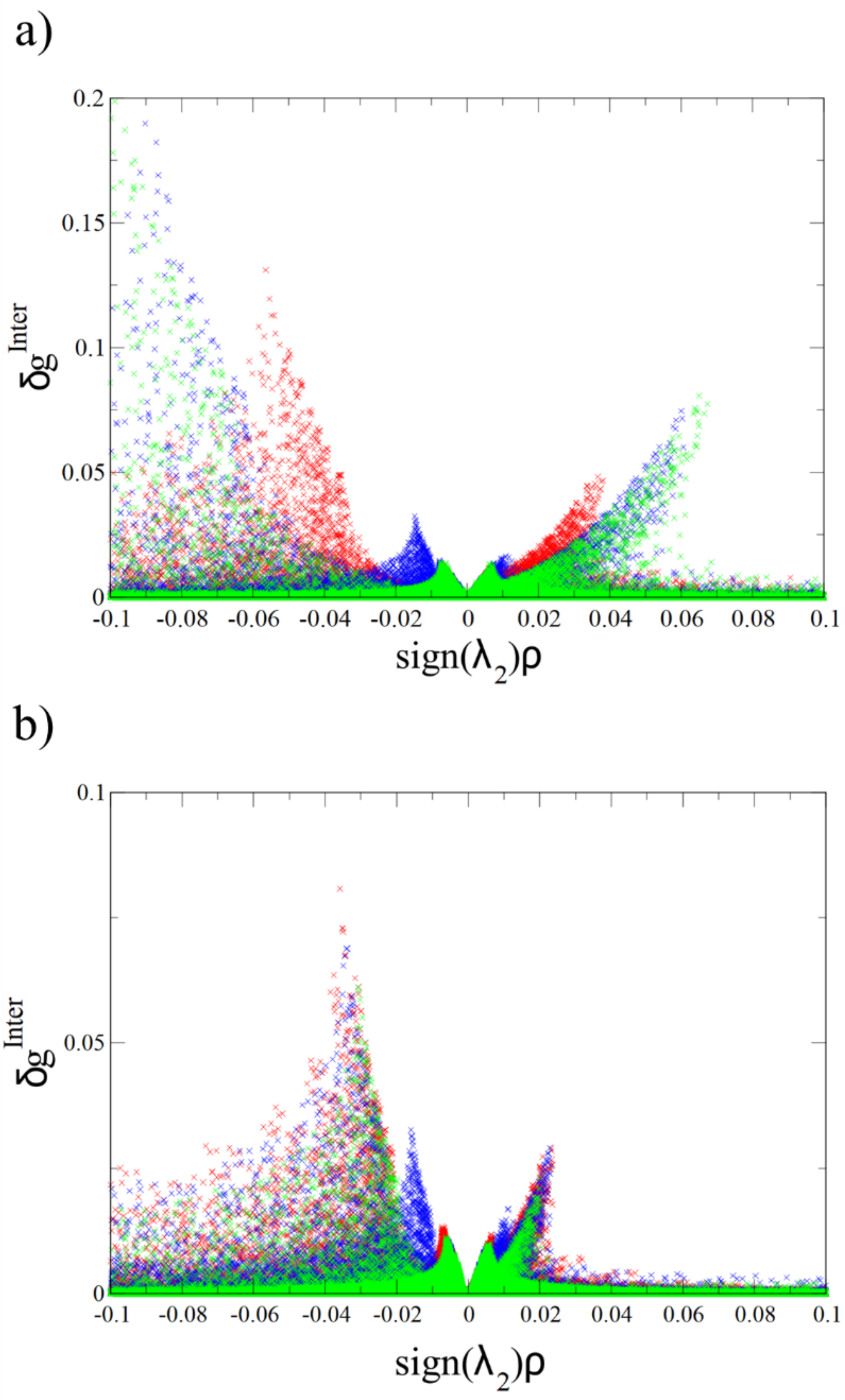

Figure S4 The δg^{inter} descriptor graph calculated for a) Type 4 clusters (Figure 5a-c): NH_3-PtPPH-H_2O---N7-mG (red), NH_3-PtPPH-H_2O---N7-mA (blue) and NH_3-PtPPH-H_2O---N1-mA (green). b) Type 5 (Figure5d-f): mG-N7---NH_3-PtPPH-H_2O (red), mA-N7---NH_3-PtPPH-H_2O (blue) and mA-N1---NH_3-PtPPH-H_2O (green).

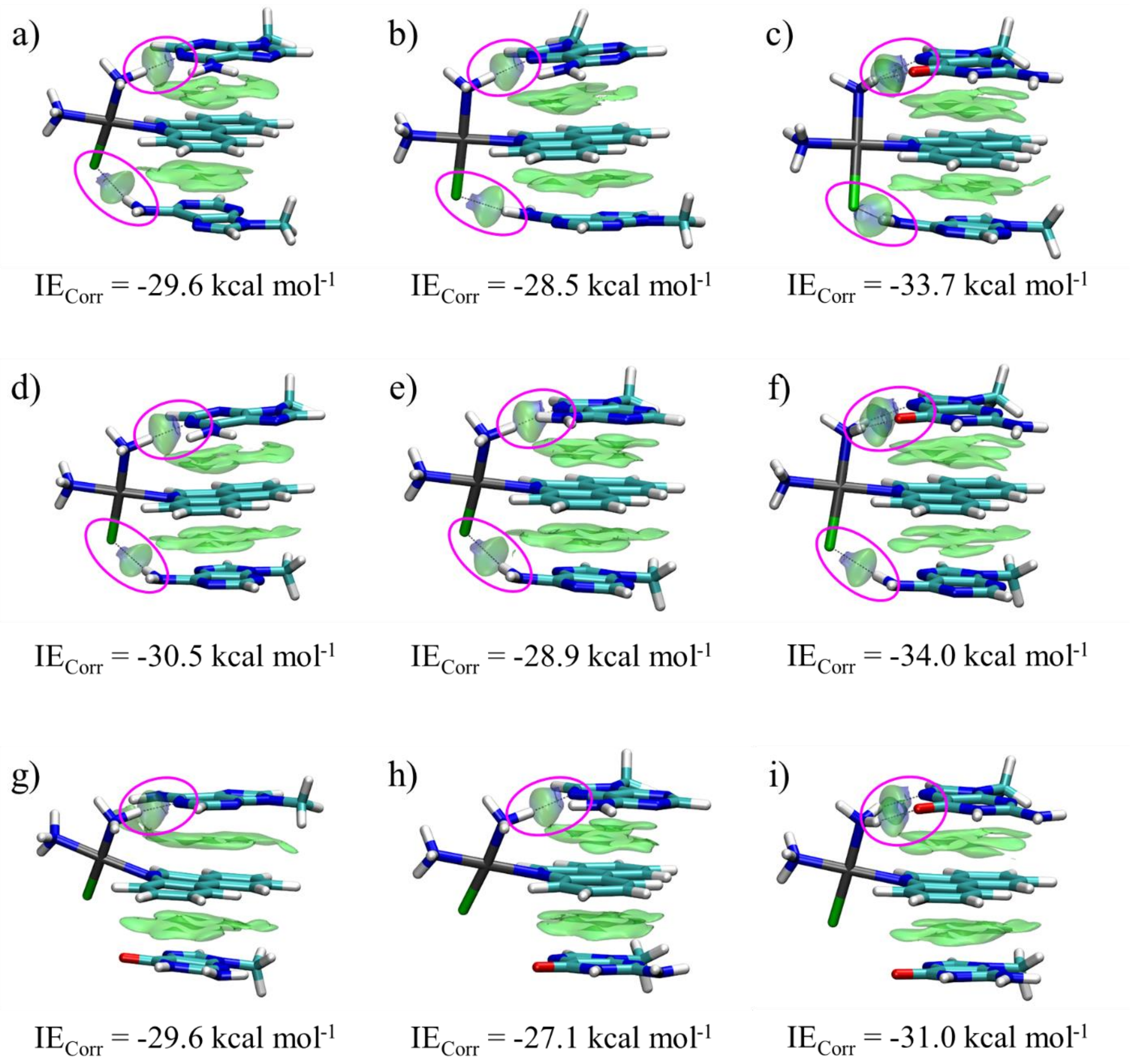

Figure S5 Optimized structures of Type 6 model: a) mA-N1---PtPPH-Cl---N1-mA; b) mA-N7---PtPPH-Cl---N1-mA; c) mG-N7---PtPPH-Cl---N1-mA; d) mA-N1---PtPPH-Cl---N7-mA; e) mA-N7---PtPPH-Cl---N7-mA; f) mG-N7---PtPPH-Cl---N7-mA; g) mA-N1---PtPPH-Cl---N7-mG; h) mA-N7---PtPPH-Cl---N1-mA; i) mG-N7---PtPPH-Cl---N7-mG. Green surfaces: $\delta g^{inter}(\rho)$ plots (isovalue 0.0055 a.u). HBs are highlighted in purple.

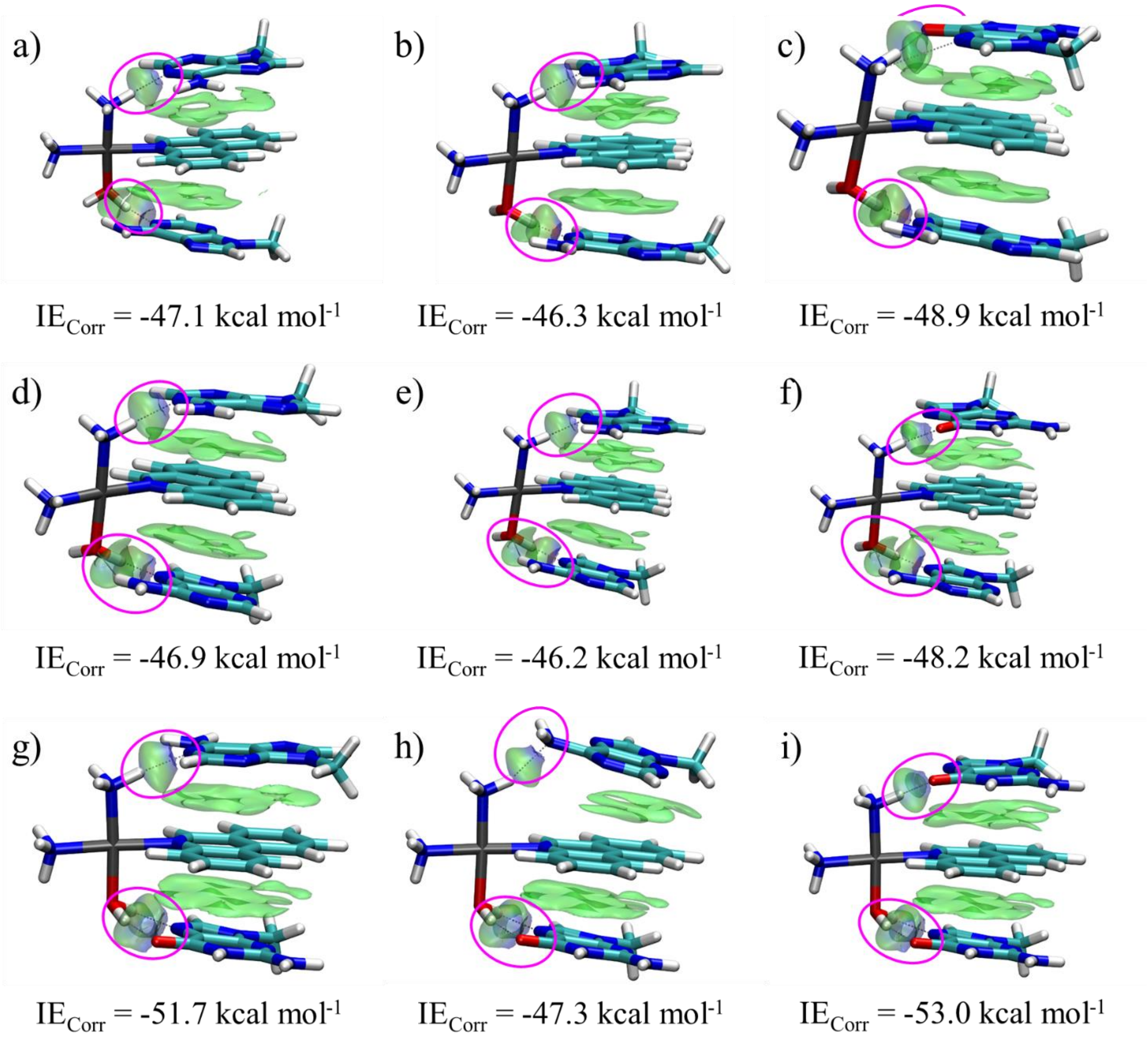

Figure S6 Optimized structure of Type 6 clusters for *aqua*-PtPPH: a) mA-N1---PtPPH-H_2O---N1-mA; b) mA-N7---PtPPH-H_2O---N1-mA; c) mG-N7---PtPPH-H_2O---N1-mA; d) mA-N1---PtPPH-H_2O---N7-mA; e) mA-N7---PtPPH-H_2O---N7-mA; f) mG-N7---PtPPH-H_2O---N7-mA g) mA-N1---PtPPH-H_2O---N7-mG h) mA-N7---PtPPH-H_2O---N7-mG; i) mG-N7---PtPPH-H_2O---N7-mG. In green $\delta g^{inter}(\rho)$ plot (isovalue 0.0055 a.u.). HBs are highlighted in purple.

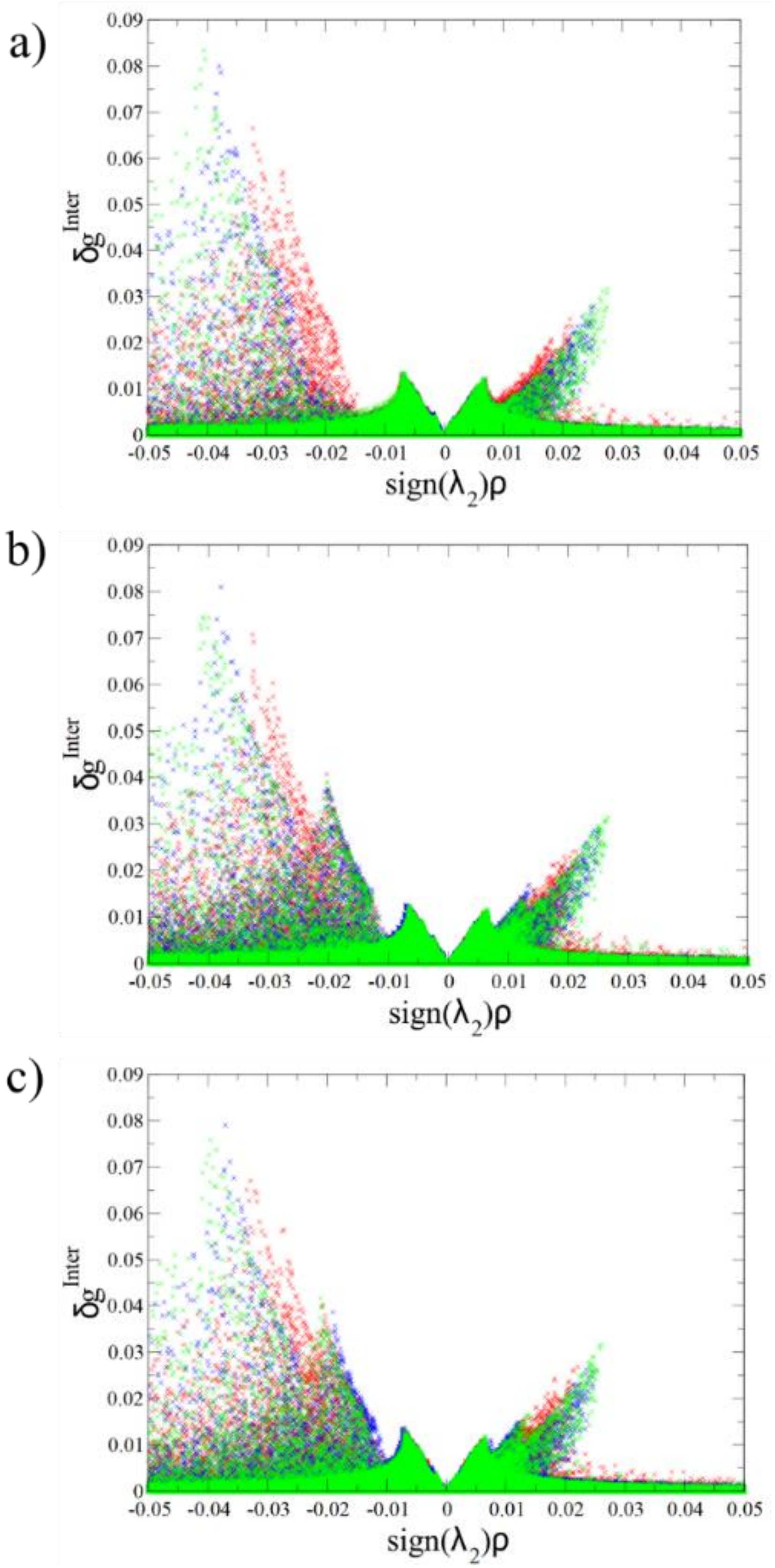

Figure S7 δg^{inter} descriptor graph calculated for Type 6 model (Figure S5): a) mG-N7---NH_3-PtPPH-Cl---N7-mG (red), mA-N7---NH_3-PtPPH-Cl---N7-mG (blue), mA-N1---NH_3-PtPPH-Cl---N7-mG (green); b) mG-N7---NH_3-PtPPH-Cl---N7-mA (red), mA-N7---NH_3-PtPPH-Cl---N7-mA (blue), mA-N1---NH_3-PtPPH-Cl---N7-mA (green); c) mG-N7---NH_3-PtPPH-Cl---N1-mA (red), mA-N7---NH_3-PtPPH-Cl---N1-mA (blue), mA-N1---NH_3-PtPPH-Cl---N1-mA (green).

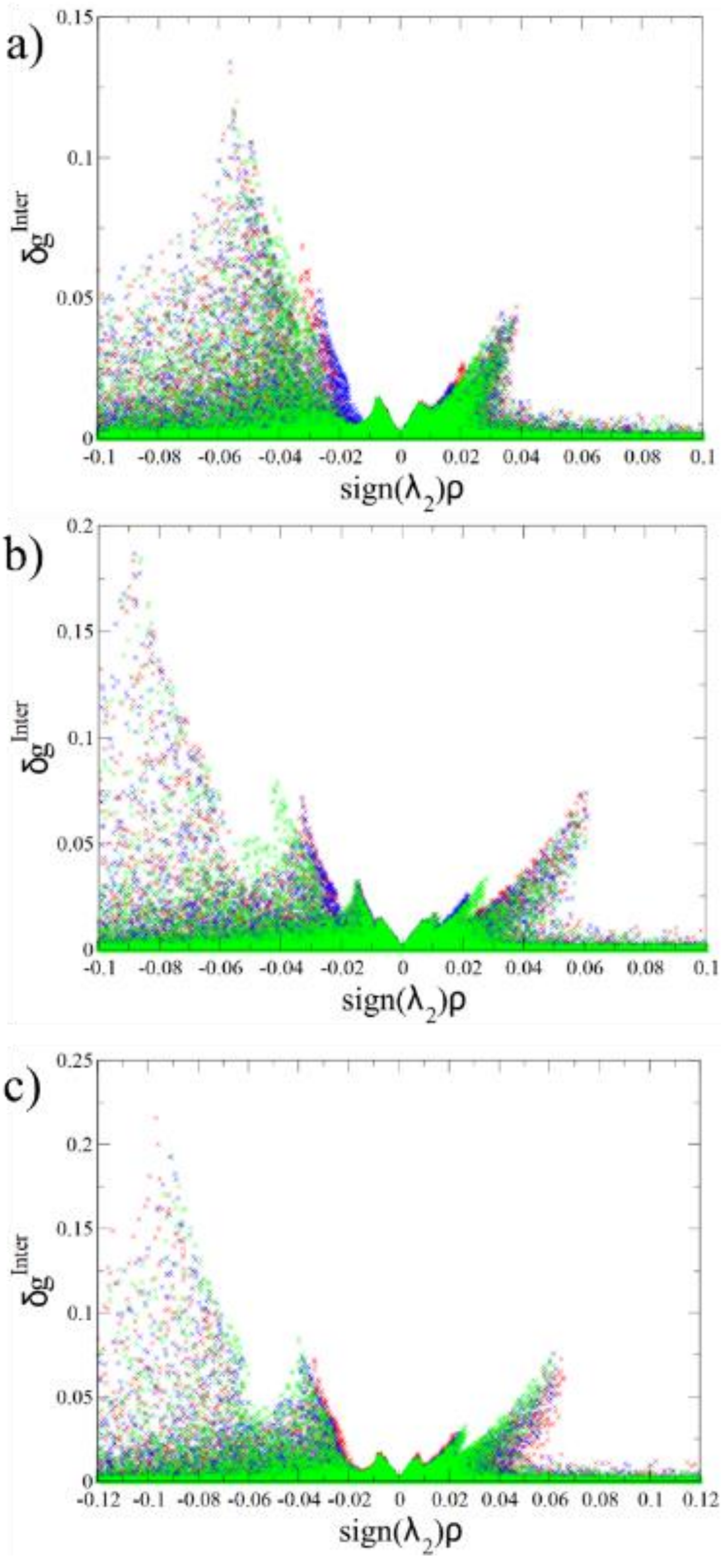

Figure S8 δg^{inter} graph calculated for Type 6 models for *aqua*-PtPPH (Figure S6): a) mG-N7---NH_3-PtPPH-H_2O---N7-mG (red), mA-N7---NH_3-PtPPH-H_2O---N7-mG (blue), mA-N1---NH_3-PtPPH-H_2O---N7-mG (green); b) mG-N7---NH_3-PtPPH-H_2O---N7-mA (red), mA-N7---NH_3-PtPPH-H_2O---N7-mA (blue), mA-N1---NH_3-PtPPH-H_2O---N7-mA (green); c) mG-N7---NH_3-PtPPH-H_2O---N1-mA (red), mA-N7---NH_3-PtPPH-H_2O---N1-mA (blue), mA-N1---NH_3-PtPPH-H2O---N1-mA (green).

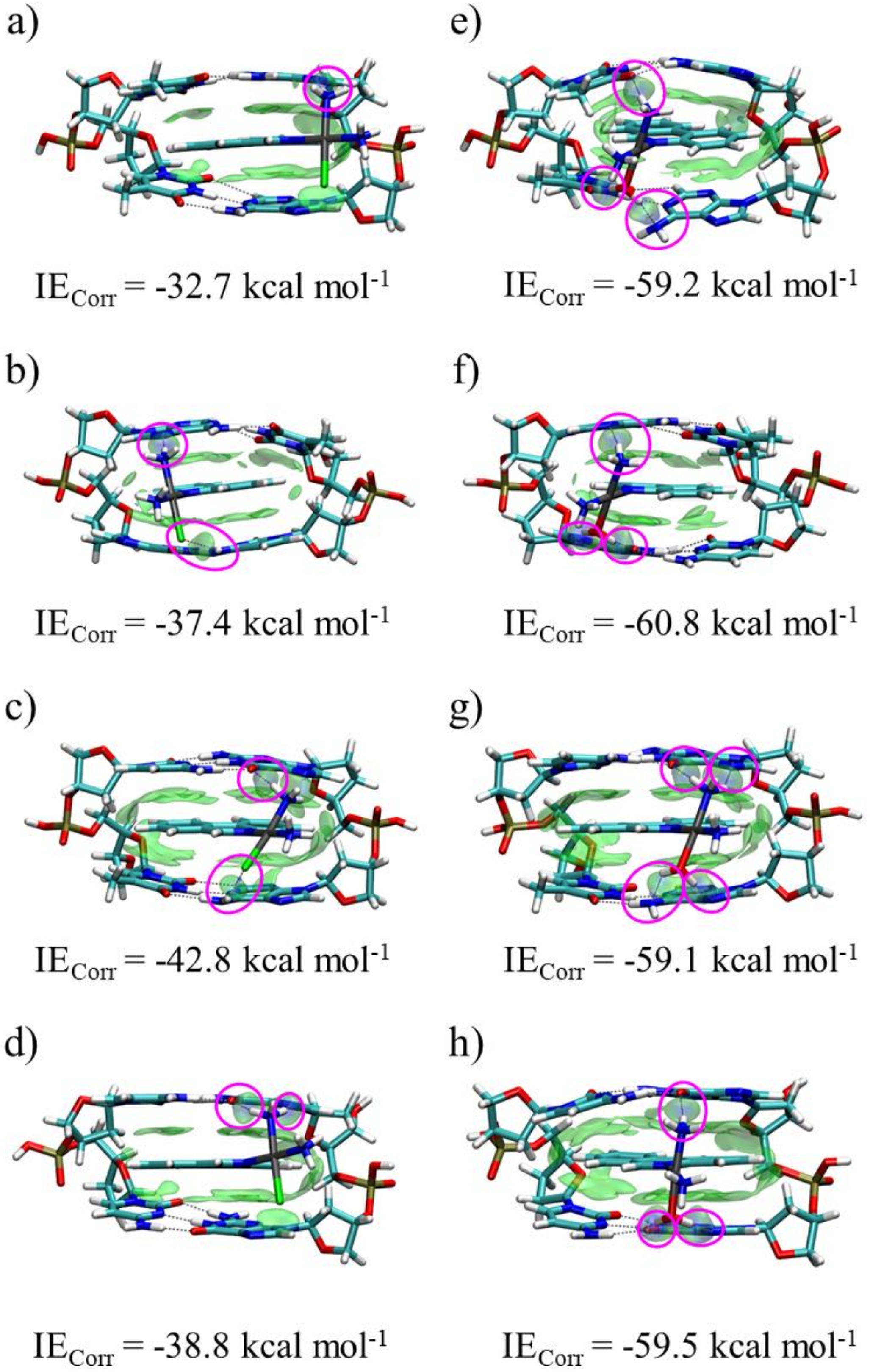

Figure S9 Optimized structures for Type 7 models: a) A-N7---PtPPH-Cl---N7-A; b) A-N7---PtPPH-Cl---N7-G; c) G-N7---PtPPH-Cl-N7-A7; d) G-N7---PtPPH-Cl---N7-G; e) A-N7---PtPPH-H_2O---N7-A; f) A-N7---PtPPH-H_2O---N7-G; g) G-N7---PtPPH-H_2O---N7-A; h) G-N7---PtPPH-H_2O---N7-G. In green color-filled $\delta g^{inter}(\rho)$ plot (isovalue 0.0055 a.u.). HBs are highlighted in purple.

Table S3. IE, IE_{Corr} and BSSE in kcal mol^{-1} in gas phase and IE in water for PtPPH and *aqua*-PtPPH in Type 7 DNA models (Figure S9).

**Sequence**	**Gas Phase**			**Water**
	**IE**	**IE_{Corr}**	**BSSE**	**IE**
PtPPH				
A-N7--- PtPPH ---N7-A	-63.1	-48.1	15.0	-47.7
A-N7--- PtPPH ---N7-G	-74.5	-59.1	15.4	-52.8
G-N7--- PtPPH ---N7-A	-92.4	-72.7	19.7	-62.5
G-N7--- PtPPH ---N7-G	-83.9	-67.0	16.9	-55.8
acqua-PtPPH				
A-N7--- PtPPH ---N7-A	-137.2	-118.9	18.3	-77.6
A-N7--- PtPPH----N7-G	-147.3	-128.3	19.0	-79.8
G-N7--- PtPPH ---N7-A	-151.8	-131.2	20.6	-79.6
G-N7--- PtPPH ---N7-G	-153.4	-131.7	21.7	-81.2

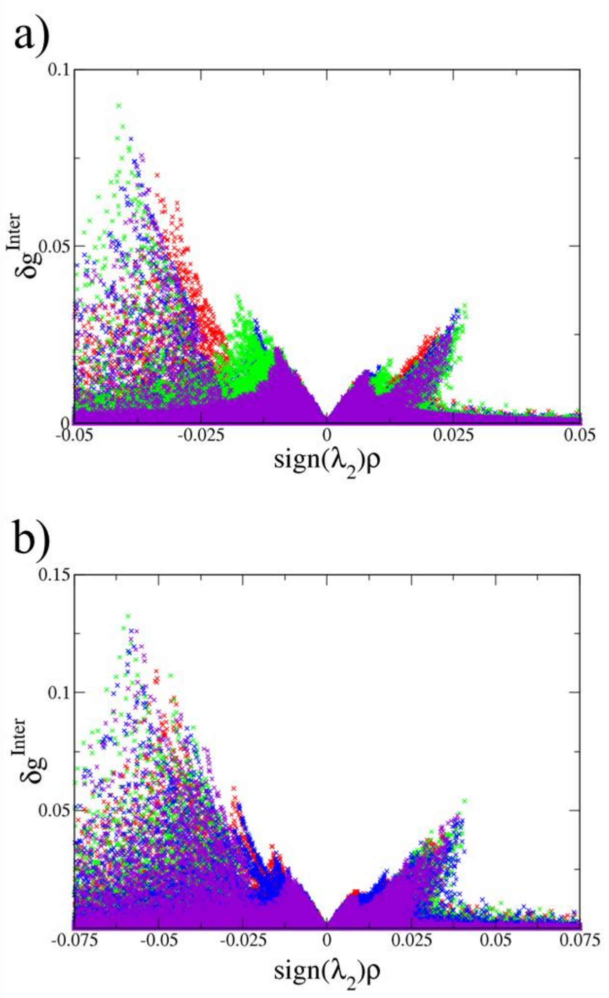

Figure S10 δg^{inter} descriptor graph calculated for Type 7 models: a) G-N7---NH_3-PtPPH-Cl-N7---G (red), A-N7---NH_3-PtPPH-Cl-N7--G (blue) G-N7---NH_3-PtPPH-Cl-N7---A (green) and A-N7---NH_3-PtPPH-Cl-N7---A (purple); b) G-N7--

-NH_3-PtPPH-H_2O-N7---G (red), A-N7---NH_3-PtPPH-H_2O---N7-G (blue) G-N7---NH_3-PtPPH-H_2O---N7-A (green) and A---N7-NH_3-PtPPH-H_2O---N7-A (purple). (Figure S9)

Table S4 Activation ($\Delta G^{\ddagger}$) and reaction (ΔG_r) Gibbs free energy (kcal mol^{-1}) for the various hydrolysis reactions considered (Figure 4). Calculated rate constants (*k*, s^{-1}) at 298.15 K are also reported.

Hydrolysis reaction	$\Delta G^{\ddagger}$	*k*	ΔG_r
isolated PtPPH in PCM water	24.7	$4.9 \cdot 10^{-6}$	10.1
isolated PtPPH in PCM water + 5 explicit water	24.1	$1.3 \cdot 10^{-5}$	12.3
Type 6 cluster	15.2	$4.5 \cdot 10^{1}$	1.0
Type 7 cluster	18.7	$1.2 \cdot 10^{-1}$	5.1
